# An Amorphous Phase Precedes Crystallization: Unraveling the Colloidal Synthesis of Zirconium Oxide Nanocrystals

**DOI:** 10.1021/acsnano.3c02149

**Published:** 2023-04-24

**Authors:** Rohan Pokratath, Laurent Lermusiaux, Stefano Checchia, Jikson Pulparayil Mathew, Susan Rudd Cooper, Jette Katja Mathiesen, Guillaume Landaburu, Soham Banerjee, Songsheng Tao, Nico Reichholf, Simon J. L. Billinge, Benjamin Abécassis, Kirsten M. Ø. Jensen, Jonathan De Roo

**Affiliations:** † Department of Chemistry, University of Basel, Mattenstrasse 24a, 4058 Basel, Switzerland; ‡ ENSL, CNRS, Laboratoire de Chimie UMR 5182, 46 allée d’Italie, 69364 Lyon, France; § ESRF Synchrotron, ID15A Beamline, 71 Avenue des Martyrs, CS40220, 38043 Grenoble, France; ∥ Department of Chemistry, University of Copenhagen, Universitetsparken 5, 2100 Copenhagen Ø, Denmark; ⊥ Department of Physics, Technical University of Denmark, Fysikvej Bldg. 312, 2800 Kgs. Lyngby, Denmark; # Deutsches Elektronen-Synchrotron DESY, Notkestraße 85, 22607 Hamburg, Germany; ¶ Applied Physics and Applied Mathematics Department, Columbia University, New York, New York 10027, United States

**Keywords:** nucleation, growth, nanoparticle, total scattering, small-angle X-ray scattering, ZrO_2_

## Abstract

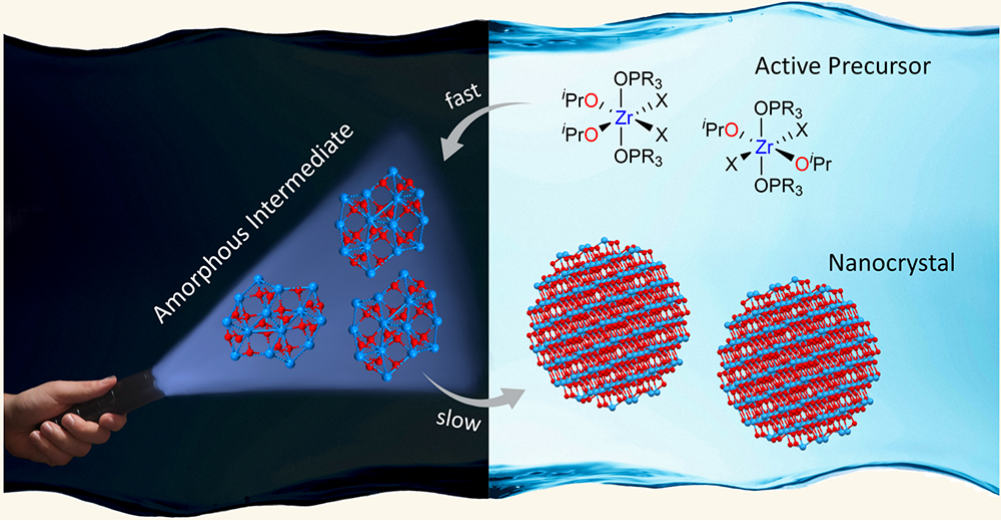

One can nowadays readily generate monodisperse colloidal nanocrystals, but the underlying mechanism of nucleation and growth is still a matter of intense debate. Here, we combine X-ray pair distribution function (PDF) analysis, small-angle X-ray scattering (SAXS), nuclear magnetic resonance (NMR), and transmission electron microscopy (TEM) to investigate the nucleation and growth of zirconia nanocrystals from zirconium chloride and zirconium isopropoxide at 340 °C, in the presence of surfactant (tri-*n*-octylphosphine oxide). Through E1 elimination, precursor conversion leads to the formation of small *amorphous* particles (less than 2 nm in diameter). Over the course of the reaction, the total particle concentration decreases while the concentration of nanocrystals stays constant after a sudden increase (nucleation). Kinetic modeling suggests that amorphous particles nucleate into nanocrystals through a second order process and they are also the source of nanocrystal growth. There is no evidence for a soluble monomer. The nonclassical nucleation is related to a precursor decomposition rate that is an order of magnitude higher than the observed crystallization rate. Using different zirconium precursors (e.g., ZrBr_4_ or Zr(O*t*Bu)_4_), we can tune the precursor decomposition rate and thus control the nanocrystal size. We expect these findings to help researchers in the further development of colloidal syntheses.

## Introduction

Sensitive structure–property relationships are at the center of nanotechnology.^1,2^ Nanocrystal structure includes shape, size (and size distribution at the ensemble level), elemental composition (including dopants and core/shell architectures), and surface chemistry. Bottom-up, surfactant-assisted synthesis has enabled structural control, albeit usually achieved by trial-and-error.^3−5^ Understanding how nanocrystals nucleate and grow would greatly advance synthetic development. A central question is how to obtain monodisperse nanocrystals with controlled size.

A surfactant-assisted synthesis of nanocrystals is a complex sequence of reactions that usually starts with the conversion of molecular precursors (P).^6^ It is often hypothesized that the precursor conversion results in a single unit of the final material: the *monomer* (M), see eq 1. This intermediate crystallizes into nanocrystals (NCs) through a process of nucleation and growth. Multiple theories exist pertaining to both nucleation and growth.
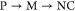
1Classical nucleation theory considers the monomer in thermodynamic equilibrium with the formed particles.^7^ The activation energy for nucleation depends on the surface energy of the particles, the temperature, and the supersaturation ratio. Studying sulfur sols, Lamer hypothesized that monomers build up in solution, reaching a critical supersaturation, and at this point, particles homogeneously nucleate (infinitely fast).^8,9^ This *burst nucleation* is mostly a hypothesis and was recently disproven in the case of Pd, InP, CdSe, and PbS(e) nanocrystals by Small Angle X-ray scattering (SAXS) and UV–vis spectroscopy.^10−15^ Instead, *continuous nucleation* was observed, evidenced by a continuous increase of the particle concentration during the reaction. Continuous nucleation is also the first step in the Finke–Watzky two-step model, although nucleation is considered an irreversible process in FW theory.^16^ Interestingly, an order–disorder equilibrium (between an amorphous and crystalline state) was observed by *in situ* TEM during the nucleation of Au nanocrystals, indicating that crystallization is reversible (under those conditions).^17^ There were more nonclassical nucleation pathways proposed. For example, the surfactant-free aqueous synthesis of calcium phosphate and magnetite involves amorphous prenucleation clusters.^18,19^ In the surfactant-assisted synthesis of Ni nanocrystals, liquid cell TEM revealed again an amorphous phase.^20^ SAXS revealed amorphous agglomerates in the synthesis of CsPbBr_3_ nanocrystals.^21^ Furthermore, magic-sized clusters with a well-defined structure have been identified in the synthesis of InP nanocrystals as intermediate species between precursors and nanocrystals.^22^ Finally, the conversion of iron oleate in iron oxide nanocrystals happens without nucleation. Iron oleate is an iron oxo cluster with three iron atoms connected by a μ_3_-oxygen atom.^23^ Esterification of the oleate leads to continuous growth of the precursor into iron oxide nanocrystals, without a separate nucleation step.

Growth models can be constructed from thermodynamic principles where monomers are incorporated into nanocrystals via either diffusion-limited growth or reaction-limited growth.^24^ While the former is size-focusing (narrowing the size distribution during growth), it appears insufficient to reconcile the monodisperse end product with continuous nucleation. A stronger focusing mechanism is found by assuming an intrinsic size-dependent growth rate, where small particles grow faster than large particles.^10,11,25^ Next, ripening is a growth mechanism where some particles dissolve to provide the necessary monomers for the growth of other particles. Some thermodynamic models consider the final nanocrystal size as an equilibrium product, the size of which is determined by the amount and type of surfactant.^26^ In the case of MnO and CdSe nanocrystals, the number of particles decreased over the course of the reaction while the size distribution narrowed (self-focusing).^27,28^ Finally, oriented attachment and coalescence are two growth mechanisms that do not involve growth by monomer.^29,30^ For example, oriented attachment was observed for ZnO and TiO_2_,^31−33^ in the absence of ligands, or for ZnS in oleylamine, a weakly bound ligand.^34^ Coalescence was shown for Ir and Pt nanocrystals, again with weakly binding ligands.^35,36^ Especially in the case of Pt, a detailed TEM analysis showed an initial increase in the number of particles and a subsequent decrease, mostly caused by coalescence and to a minor extent by particle dissolution.^36^ Some particles grew by monomer/precursor attachment and others by coalescence. While regular growth immediately formed single-crystalline particles, a polycrystalline particle was initially observed after coalescence. Structural rearrangement took about 16 s after coalescence to produce a single crystalline nanocrystal. Irrespective of the mechanism, the nanocrystals stop growing after reaching a certain size (i.e., self-focusing).

**Figure 1:**
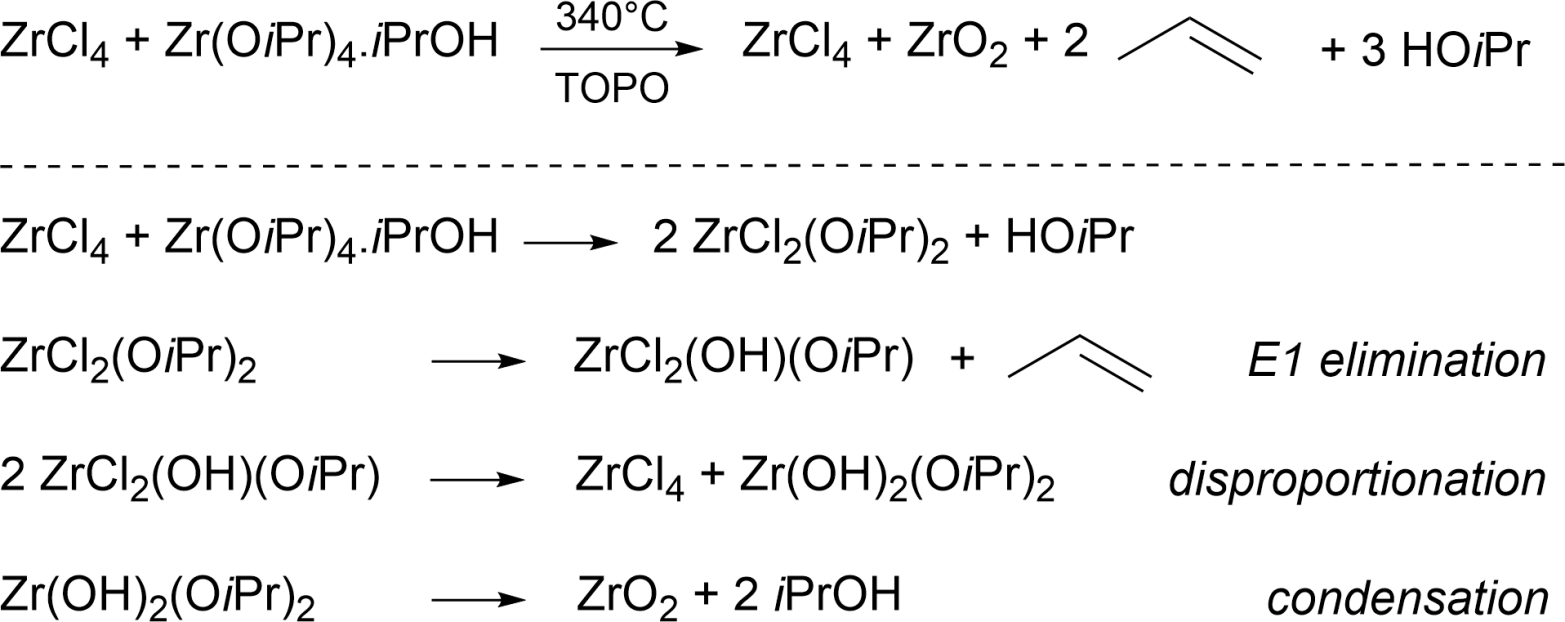
Synthesis of zirconia from zirconium chloride and zirconium isopropoxide isopropanol complex at 340 °C in trioctylphosphine oxide (TOPO). The chemical mechanism involves first the formation of mixed chloro-alkoxide species, which undergo E1 elimination, disproportionation, and condensation reactions.^40^

Compared to the exhaustively studied semiconductor and metallic systems, little is known about the surfactant-assisted synthesis of group 4 metal oxides. Previous studies only focused on surfactant-free hydrothermal syntheses, where an amorphous material rapidly forms before crystallizing into highly aggregated particles.^37,38^ Surfactants are generally hypothesized to control and slow down the precipitation rates. For example, highly monodisperse and colloidally stable zirconia nanocrystals are synthesized from zirconium chloride and zirconium isopropoxide (complexed with isopropanol) at 340 °C in trioctylphosphine oxide (TOPO).^39^ We recently showed that a mixed chloroalkoxide precursor species is formed, which undergoes (predominantly) E1 elimination to zirconium hydroxide and propene, see Figure 1.^40^ After disproportionation and condensation, zirconium chloride, zirconia, and isopropanol are formed as products. The ZrCl_4_ coproduct is retrieved as a Lewis acid–base complex with two TOPO ligands,^40^ bringing the coordination number of zirconium to six. The nanocrystals are covered with TOPO, TOPO’s decomposition products, and chloride.^41^ There is no information about their nucleation and growth mechanism. Understanding these processes might help to further control the nanocrystal size, introduce dopants and access the core–shell structures of group 4 oxides. Since also titania and hafnia nanocrystals can be synthesized by similar routes, the zirconia model system is relevant for the entire group 4.^42−44^ Within this context of group 4 oxides, a Lewis structure of a MO_2_ “monomer” is hard to conceive. A slightly more realistic monomer could be, e.g., Zr(OH)_4_, but group 4 metal hydroxide species are highly reactive and usually already condense before the tetrahydroxide species can be fully formed.

Here, we investigate the crystallization mechanism of zirconia nanocrystals in TOPO, synthesized from zirconium halide (chloride or bromide) and zirconium isopropoxide. We combine X-ray pair distribution function analysis, small-angle X-ray scattering, nuclear magnetic resonance, and transmission electron microscopy to investigate their nucleation and growth. We determined the temporal evolution of the number of particles, the particle size, the size distribution and the yield. We find that after an initial fast increase in the number of particles, the total particle number drops with a concomitant reduction of the size dispersion. However, the number of crystalline particles remains constant after an initial rapid nucleation event. We established the mass balance of the reaction and show that amorphous particles are intermediate en route to the final nanocrystals. Finally, we demonstrate how precursor conversion kinetics controls the nanocrystal size.

## Results and Discussion

We synthesize zirconia nanocrystals at 340 °C, from a 1:1 molar mixture of Zr(O*i*Pr)_4_·*i*PrOH and either ZrCl_4_ or ZrBr_4_. The synthesis is performed in pure trioctylphosphine oxide (TOPO), which acts as both ligand and solvent. Details of the precursor chemistry are shown in Figure 1.

### Small Angle X-ray Scattering Analysis

**Figure 2:**
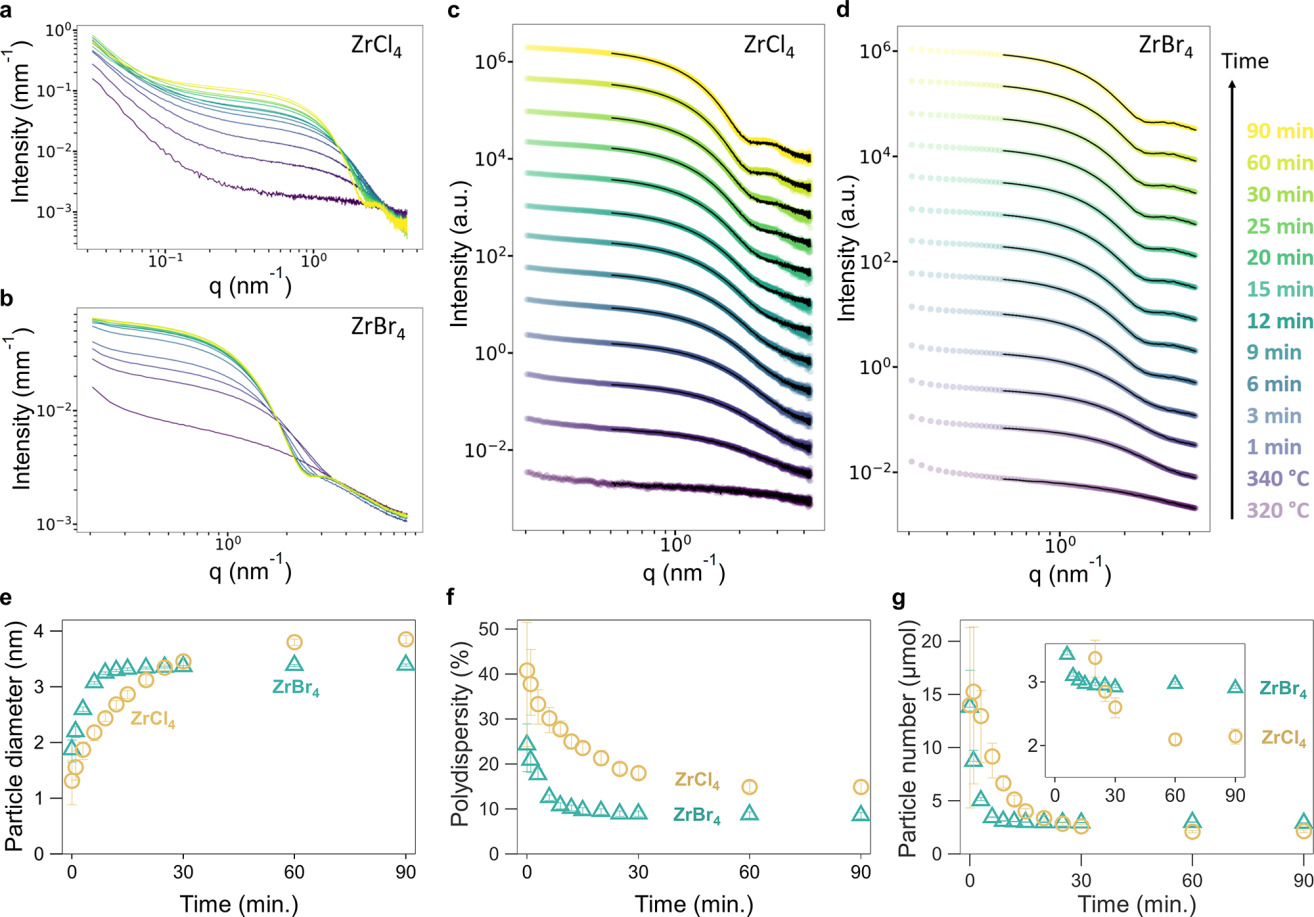
*Ex situ* SAXS analysis on reaction aliquots. Normalized SAXS pattern (a,b) and corresponding fits (c,d) for reaction mixtures with either ZrCl_4_ or ZrBr_4_ as halide source. The different patterns are shifted with respect to one another for better visualization. Time evolution at 340 °C for the particle diameter (e), the polydispersity (f), and the particle concentration (g) obtained after fitting the experimental data.

Given the success of small-angle X-ray scattering (SAXS) in delivering insights into nucleation and growth mechanisms,^10,11,14,15,21^ we also take it here as a starting point in our investigation. We performed *ex situ* SAXS measurements on aliquots, taken from the reaction mixture at 300 °C, 320 °C, and 340 °C during the heating ramp, and at different time intervals at 340 °C. The samples were diluted in toluene and measured in a flow-through setup to obtain signals in absolute intensity (Figure 2a,b), and to estimate the particle concentration and size (see Experimental Section and Supporting Information for details).^45^ Qualitatively, the evolution of the scattering curves suggests the formation of spherical particles for both reactions (with either ZrCl_4_ or ZrBr_4_). With increasing reaction time, the signal increasingly resembles the form factor of a sphere, with a plateau at intermediate *q* and an abrupt decrease followed by oscillations for *q* larger than 2 nm^–1^. Both the increasing intensity of the plateau and the shift of the position of the abrupt decrease toward smaller *q*, indicate particles growing in size. The increasing amplitude of the oscillations suggests that the polydispersity decreases with time.

The particle size and concentration were quantified by fitting the normalized scattering curves. At lower *q*, we observe an increase in the intensity (Figure 2a,b), which probably results from a partial aggregation of the particles. This would require introducing a structure factor in the fit. To minimize this effect and to consider a model of independent scatterers, our experimental data are fitted in the *q* region larger than 0.5 nm^–1^. The SAXS patterns contain contributions from precursors and zirconia particles in solution:
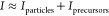
To approximate the signal of the precursors, we use the experimental SAXS signal of the ZrCl_4_ reaction mixture, aliquoted at 300 °C. At this temperature, precursor conversion has not yet started.^40^ Therefore, our fitting function becomes the sum of the scattering cross section of a distribution of polydisperse spheres and the experimental precursor signal, multiplied by a fitting parameter (*F*) set between 0 and 1:
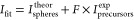
This approach leads to very good fits for all the SAXS patterns (Figure 2c,d), allowing us to derive the particle radius, polydispersity and concentration during the synthesis, for both systems (Figure 2e–g).

For both reactions (with either ZrCl_4_ or ZrBr_4_ as halide source), there are a lot of particles present in the solution once the reaction temperature reaches 340 °C. We observe particle growth (increase in diameter) with a simultaneous reduction in the polydispersity (Figure 2e,f). The higher polydispersity in the first 10 min is also responsible for the higher error in the diameter value. The final particle size obtained in the ZrCl_4_ reaction (3.9 nm) is higher than for the ZrBr_4_ reaction (3.4 nm), consistent with previous reports.^39^ The particles in the chloride reaction mixture keep growing for 90 min, while the growth of the bromide particles stops after about 15 min. Interestingly, the particle number *decreases* over the course of the reaction. These results stand in stark contrast to many other surfactant-assisted nanocrystal syntheses, which featured a continuous *increase* of the particle concentration (continuous nucleation).^10−14^ Our data thus rather suggest a coalescence or ripening mechanism.^27,28,36^ A lot of particles nucleate at the start and grow at the expense of other particles. When comparing the two halide sources, the particles from the bromide reaction grow faster, have a lower dispersity, are smaller in size and their final concentration is higher. For the same material yield, one can have either a few, large particles or many, small particles. The particle concentration at the end of the bromide reaction is indeed about 50% higher than in the chloride reaction (Figure 2g), consistent with their diameter difference.

### X-ray Pair Distribution Function Analysis

**Figure 3:**
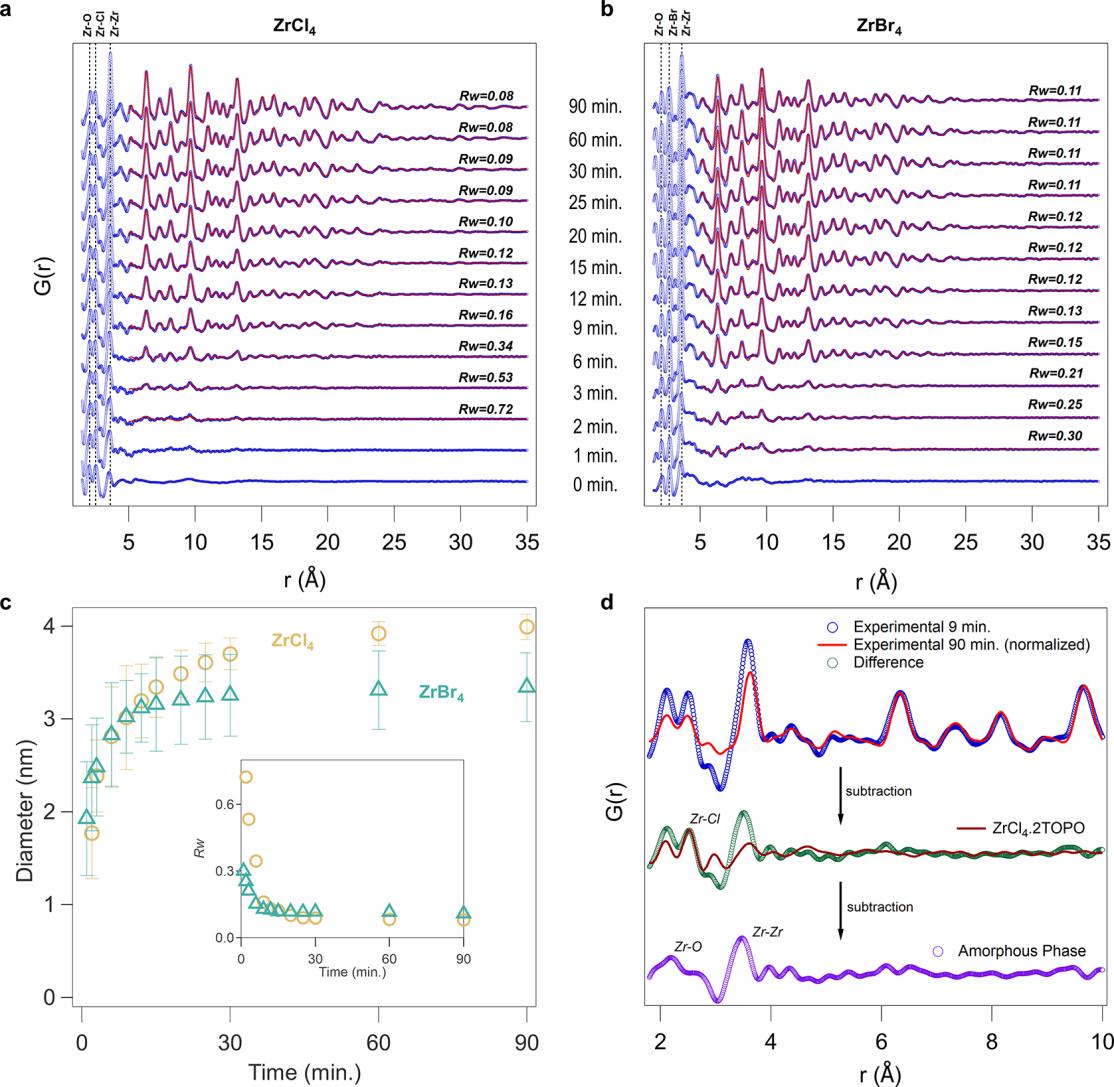
*Ex situ* PDF analysis on reaction aliquots. The experimental (blue circles) and calculated (red line) PDF for the reaction aliquots with either ZrCl_4_ (a) or ZrBr_4_ (b) as halide source. The goodness of fit is indicated by *Rw*. The reaction temperature of 340 °C is reached at *t* = 0 min. (c) The refined nanocrystal size by PDF and corresponding *Rw* values (inset) of the reaction aliquots. The refined parameters are shown in Table S1–S2. (d) Extraction of PDF pattern of the amorphous intermediate phase for the 9 min aliquot (ZrCl_4_ reaction). The blue circles represent the measured PDF of the aliquot, and the red line corresponds to the normalized PDF of the reaction crude product (90 min aliquot). After subtraction of the two, the data in the green circles are obtained. The experimental PDF of the ZrCl_4_·2TOPO byproduct (brown line) is scaled and subtracted from the green data, obtaining the PDF of the amorphous intermediate (violet circles).

X-ray total scattering and pair distribution function (PDF) analysis are great tools for understanding the chemistry of nucleation.^46^ In our previous work, focusing on the precursor chemistry of this reaction, we used PDF analysis to confirm the chloroalkoxide precursor and the ZrCl_4_·2TOPO byproduct.^40^ At room temperature, the PDF of the chloride reaction mixture features three interatomic distances, Zr–O (2.0 Å), Zr–Cl (2.5 Å), and Zr–P (3.5 Å), as expected for the ZrCl_2_(O*i*Pr)_2_·2TOPO precursor species. These atom pairs can still be recognized in the PDF of the first aliquot taken at 340 °C, see Figure 3a (0 min). We observe similar atomic pair distances for the reaction mixture with ZrBr_4_, where the Zr–Br distance is observed at 2.7 Å (Figure 3b).

Within minutes after reaching 340 °C, zirconia crystals start to form and grow in size, indicated by the appearance of correlations at higher distances (Figure 3a,b). The Zr–Cl and Zr–Br distances contract slightly but remain present until the end of the reaction and are assigned to the TOPO complexes of ZrCl_4_ and ZrBr_4_. For the chloride reaction, this complex is ZrCl_4_·2TOPO. For the bromide reaction, the exact nature of the complex is unclear since the complexation between ZrBr_4_ and TOPO is not fully understood (Figure S3). Nevertheless, the ^31^P NMR spectrum at the end of the reaction is almost identical with the spectrum of ZrBr_4_ and 8 TOPO equivalents. Therefore, we conclude that the metal halide is formed as the byproduct, in both cases. We infer that the reaction with ZrBr_4_ likely follows a similar precursor conversion mechanism as the reaction with ZrCl_4_ (Figure 1). Previously, we have shown that the PDF of the last aliquot (end of reaction) can be well described by a two-phase model, featuring (i) tetragonal (*P*4_2_/*nmc*) zirconia and (ii) the ZrCl_4_·2TOPO complex.^40^ This approach does not work as well for the bromide case, since we do not have an accurate structure for the TOPO complex of ZrBr_4_. The two-phase modeling strategy also appears challenging for the early aliquots in the chloride reaction. The contribution of the crystal phase is low and the molecular zirconium precursors species are not yet fully converted to the ZrCl_4_·2TOPO byproduct.

To quantify the crystal size, we thus start the fit from 5 Å, cutting off the contributions from the molecular precursors and only focusing on the tetragonal zirconia phase, see Figure 3a,b. For the bromide case, we follow the same strategy but we add a decaying sinusoid to the model. The latter takes care of a periodic oscillation in the data. This effect may originate from the presence of organic ligands in the material.^47^ Sinusoidal oscillations can also appear in PDF measurements due to solvent restructuring.^48^ The refined particle size and goodness of fit (*Rw*) are shown in Figure 3c. In the chloride reaction mixture, we could not obtain a decent refinement for the aliquots taken at 0 and 1 min (although one can argue that very weak signals are present after 1 min). Only after 2 min we can refine a nanocrystal structure with a diameter of 1.8 nm. The crystal pattern is still fairly weak (due to a low concentration of crystals), which is reflected in the poor (high) *Rw* of the refinement. The particles grow over time to the expected crystal diameter of about 4 nm, and the *Rw* of the refinements decreases to 8%. The PDF of the bromide reaction mixture could not be refined at *t* = 0 min, even though some very weak signals are observed. After 1 min, we are able to refine the PDF reasonably well (*Rw* = 0.3) and the first observable nanocrystal is also 1.9 nm in diameter. The bromide reaction forms crystalline material faster than the chloride reaction, but the particles are growing only to about 3.3 nm in size. The bromide nanocrystals reach their final size after about 30 min, while the chloride particles keep growing for up to 90 min. This is even more obvious when the nanocrystal volume is plotted over time (Figure S4). The obtained crystal size from PDF at various time points is in agreement with the size calculated from the TEM images of purified nanocrystals (Figure S5), indicating that the particles are monocrystalline.

We also followed the reaction *in situ* in a 3 mm NMR tube heated by an aluminum metal block with heating cartridges (see Experimental Section and Figure S6). Similar trends are observed (see Figure S7), but in general, the signal-to-noise ratio is much lower compared to the *ex situ* experiments. Most importantly, we conclude that no phase transformation took place in the crystals upon quenching the reaction aliquots to room temperature.

### The Emergence of an Amorphous Phase

Upon reaching 340 °C (*t* = 0 min), SAXS reveals the presence of nanoparticles, while PDF demonstrates the absence of nanocrystals (no long-range correlations). Since SAXS detects any nanoparticle, crystalline or amorphous, we hypothesize that the first particles observed by SAXS are amorphous (i.e., disordered). To investigate this hypothesis further, we turn now to the short-range PDF data (1.5–5 Å). This complex region contains contributions from precursors, byproducts, crystalline (ordered) particles, and amorphous (disordered) particles. We first analyze the chloride reaction aliquot at *t* = 9 min (Figure 3d). We know that after 9 min there is no precursor left and the only molecular zirconium species is the byproduct; ZrCl_4_·2TOPO (Figure S8).^40^ The absence of precursors simplifies the PDF analysis. To extract the contribution of the amorphous particles, we proceed as follows.

We scale the PDF of the final aliquot (90 min) to match the intensity of the peaks beyond 5 Å (Figure 3d). As expected, a significant deviation is observed in the short *r*-range (1.5–5 Å). We then subtract the two PDFs and obtain the green data. This procedure removes the entire crystalline fraction and part of the ZrCl_4_·2TOPO byproduct. The latter is only partially removed due to the difference in the relative concentration of crystals and byproduct at the two time points. Therefore, we scale the experimental PDF of ZrCl_4_·2TOPO to the Zr–Cl peak in the green data and subsequently subtract it. We thus arrive at the residual PDF, free of crystals and byproduct. Since also precursor is absent (evidenced by NMR), the residual PDF belongs to an amorphous intermediate. We exclude the possibility of a monomer due to the pronounced Zr–Zr distance in the PDF. No long-range order is present in the PDF of the amorphous phase. The amorphous PDF signal, together with the SAXS results, firmly establish the existence of amorphous particles as reaction intermediates. By extending the analysis to other aliquots (Figure S9), we verified that, with time, the crystalline phase captures a higher proportion of the short-range structure, confirming that the amorphous phase is an intermediate that disappears over the course of the reaction. To rule out the possibility of amorphization while sampling/cooling down, similar analyses were carried out on the *in situ* data set and similar results are obtained (Figure S10).

### The Mass Balance of the Reaction

To gain further insight into the reaction mechanism and to connect the crystallization process to the precursor decomposition process, we sought to quantify all zirconium species in the reaction and have a closed mass balance. Subsequently, we aimed at determining the rate of each step. In the case of semiconductor nanocrystals, the precursor-to-monomer (P to M) conversion kinetics is assumed slower than the crystallization kinetics, see eq 2.^49,50^ A rate-limiting precursor conversion is a postulated prerequisite for controlling the size by the reaction rate.^51,52^
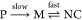
2

**Figure 4:**
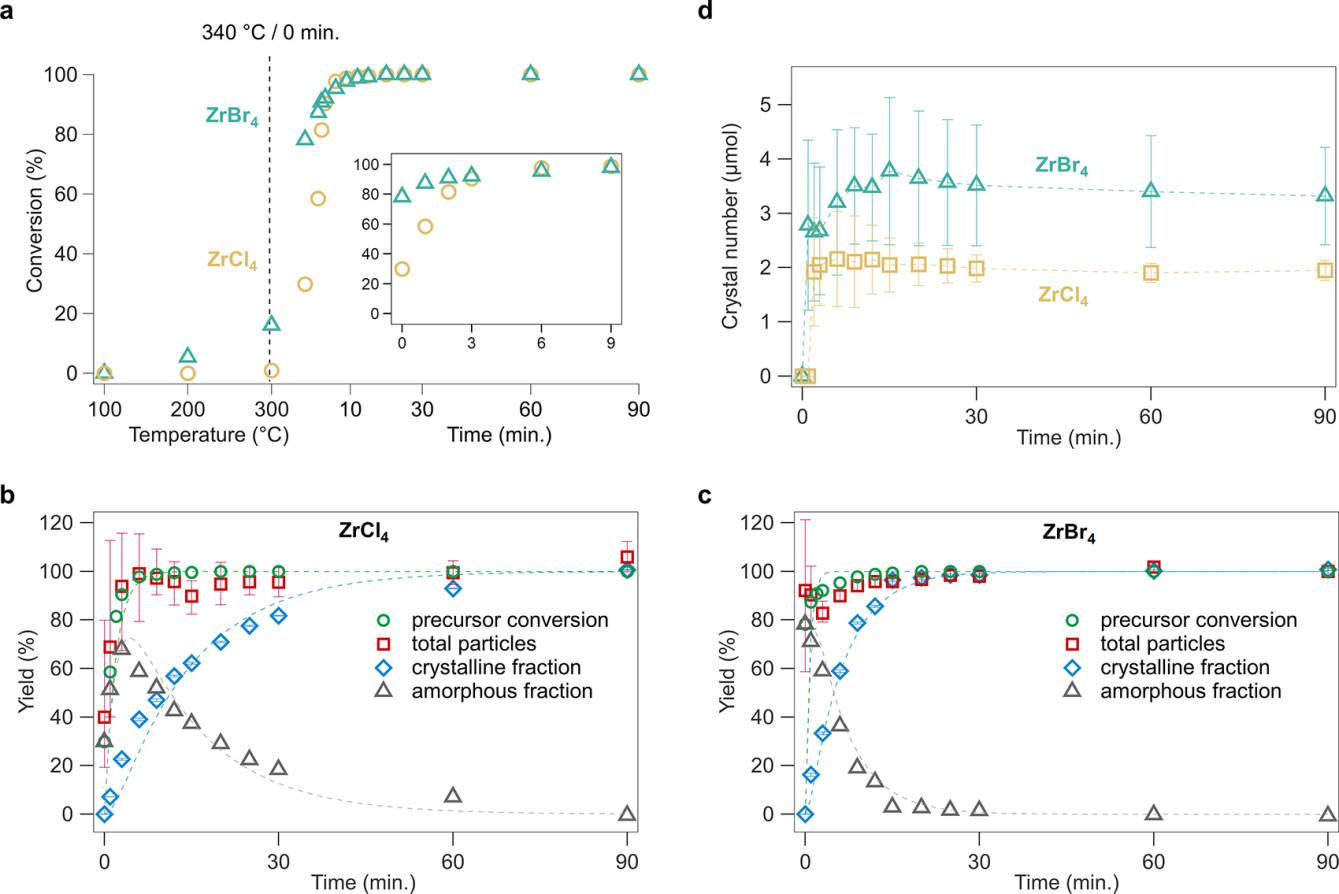
(a) The disappearance of zirconium isopropoxide moieties determined with ^1^H NMR. The inset shows more detail for the first 9 min at 340 °C. The corresponding ^1^H spectra are shown in Figures S11–S12. The yield of the reaction, from the perspective of precursor conversion, the total particles formed (from SAXS analysis), the crystalline fraction (from reciprocal total scattering analysis), and the amorphous fraction (total particles minus crystalline fraction) for reaction mixtures with either ZrCl_4_ (b) or ZrBr_4_ (c) as halide source. The dotted lines indicate the fit obtained using the simple two-step mechanism (eq 3). (d) The number of nanocrystals as a function of time, calculated from the crystalline yield and the crystal size (PDF).

To quantify the precursor conversion in our system, we followed the disappearance of the isopropoxide resonance in ^1^H NMR, see Figure 4a (corresponding ^1^H spectra are shown in Figure S11–S12). We observe faster conversion with ZrBr_4_, compared to ZrCl_4_. Even during the heating phase to 340 °C, the bromide reaction mixture starts to convert at 200 °C, while the chloride reaction only starts converting between 300 and 340 °C. Consequently, at *t* = 0 min (at 340 °C), the bromide reaction progress is already about 80%. Interestingly, the bromide reaction slows down considerably by the end of the precursor conversion. For both precursors, full conversion is achieved after 9 min at 340 °C.

In Figure 4b,c, we plot the yield of precursor conversion together with the yield of crystalline particles, amorphous particles and total particles (crystalline and amorphous). We calculated the total particle yield from the SAXS data using the particle size, the size distribution, and the particle concentration assuming the bulk density of zirconia. For the end of the reaction, we obtain a yield (*n*_ZrO_2__/*n*_Zr_) of 63% with ZrCl_4_ and 58% with ZrBr_4_, consistent with the obtained yield after isolation and purification (determined gravimetrically). We know the zirconium halide byproduct is responsible for the lower than 100% yield (based on the metal). Therefore, the unbiased results from SAXS satisfy the mass balance of zirconium according to Figure 1. To focus on the oxide formation, we rescaled the yield from SAXS to 100% at the end of the reaction, which also corresponds to 100% precursor conversion. The rate of precursor conversion is equal to the rate of particle formation since the precursor conversion data and the total particle (SAXS) data coincide, within error. This means that, at any time, all the zirconium atoms are either in the form of precursor, byproduct, or part of a particle (amorphous or crystalline). We do not find evidence for another species such as molecular solute or a “monomer”.

We calculated the yield of crystalline particles from the total scattering data, by integrating the area under a selected Bragg peak (Figure S13) after background subtraction. Also here, we scale the yield to 100% at the end of the reaction. This assumes that no amorphous phase is present at the end of the reaction. It is a fair approximation given that the PDF of the last reaction aliquot could be accurately modeled by only the crystalline phase and the zirconium chloride byproduct.^40^ We then calculate the amorphous fraction of particles by subtracting the crystalline fraction from the total particle yield. Given our conclusion that precursor conversion and total particle yield are identical, we used here the precursor conversion yield for the subtraction, since there is less scatter on this data. Finally, with crystal yield and nanocrystal size in hand, we could also calculate the concentration of nanocrystals over the course of the reaction (Figure 4d).

Focusing first on the chloride reaction in Figure 4b, one observes that the precursor conversion is complete at 9 min, but the crystalline yield is only about 50%. In addition, there is no evidence for a monomer. Hence this reaction is not well described by eq 2. Instead, precursor conversion is clearly faster than crystallization. Since the species formed by precursor conversion (metal hydroxide) is highly reactive, this leads to a buildup of amorphous intermediate (*I*). The intermediate then slowly recrystallizes into nanocrystals, see eq 3. Similar conclusions are reached for the bromide reaction although the crystallization rate appears faster, Figure 4c.
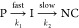
3

To obtain more quantitative insight into the reaction kinetics, we modeled the time-dependent concentration of species. Starting from the simplest model containing two elementary steps (Mechanism 3), we can reasonably well describe the kinetics of both the chloride and the bromide reaction. The resulting fit is plotted onto Figure 4b,c as dotted lines (and also plotted in Figure S14 and S20). The rate constants are provided in Table 1. The bromide crystallization rate constant (*k*_2_) is about double compared to the chloride crystallization rate constant. In both cases, the precursor conversion rate constant is about 1 order of magnitude higher than the crystallization rate constant.

**Table 1:** Comparison of Reaction Rate Constants Obtained after Fitting Various Mechanisms for Reaction Mixtures with Either ZrCl_4_ (a) or ZrBr_4_ (b) as Halide Source^a^

mechanism	precursor	*k*_1_	*k*_2_	*k*_3_
eq 3	ZrCl_4_	8.8 ± 0.2 × 10^–3^	1.2 ± 0.1 × 10^–3^	–
eq 3	ZrBr_4_	2.3 ± 0.1 × 10^–2^	2.8 ± 0.3 × 10^–3^	–
eq 4	ZrCl_4_	8.8 ± 0.2 × 10^–3^	4.7 ± 0.2	1.0 ± 0.3
eq 5	ZrBr_4_	123 ± 2	4.7 ± 0.4	17 ± 1

a The unit of rate constants are s^–1^ for first order and M^–1^ s^–1^ for second order.

Focusing further on the chloride reaction, we tested multiple other mechanisms to explore the possibility of second order reactions, equilibria, and autocatalytic mechanisms (Figure S15–S19). Taking the precursor conversion as second order, we obtain a considerably worse fit (Figure S15). This gives us further confidence in the E1 elimination mechanism since the latter should be first order. A mechanism that includes an equilibrium between precursors and intermediates does not fit significantly better than Mechanism 3 (Figure S17). The back reaction (I → P) has an almost negligible rate constant. The fit improved when taking the crystallization step as second order (Figure S16). Finally, the best agreement was obtained with Mechanism 4 (Figure S19).
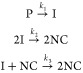
4The precursor conversion is first order, the second step is second order (a coalescence nucleation mechanism), and the third step is autocatalytic growth by the addition of the amorphous species. The rate constants are reported in Table 1. Although Mechanism 4 has elements of the Finke–Watzky (FW) mechanism,^16^ there are important differences. The FW mechanism features a slow nucleation step, directly from precursors that can be 6 orders of magnitude slower than the autocatalytic growth. Here, we find that *k*_2_ is five times higher than *k*_3_, and particles nucleate from amorphous intermediates.

We also explored different mechanisms for the bromide reaction (S21–S25). The precursor conversion reaction is not very well described by first order kinetics. The data are better described by a second order process (Figure S21), or by an equilibrium between precursor and intermediate (Figure S23). We cannot distinguish between the two scenarios based solely on the bromide data, but the chloride data indicate that the back reaction is negligible. Taking thus the precursor conversion as second order, we considered the crystallization to be second order (Figure S22) or autocatalytic (Figure S24–S25) and the best fit was found for Mechanism 5.
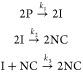
5

Interestingly, *k*_2_ is refined to the same value as for the chloride reaction, but *k*_3_ is much higher for the bromide reaction, indicating that the nucleation rate constant is similar in the bromide and chloride reactions, but that the growth rate constant is higher in the bromide reaction. Even though our simple model did not take into account differences in nanocrystal concentration, Figure 4d indicates only a 1.7-fold higher NC concentration in the bromide reaction, insufficient to explain the 17-fold rate enhancement. Differences in surface adsorption of bromide and chloride might play a role in the growth rate.^41^ Finally, the fast growth rate seems to be in contradiction with the smaller nanocrystal size in the bromide reaction.

### Nucleation and Growth Mechanism

The nucleation of nanocrystals is observed in Figure 4d. After a sudden increase, the number of crystalline particles stays constant. There is more scatter on the crystal number in the bromide case, but the error on the size is larger due to the more complex PDF refinement (oscillations modeled by sinusoid). Therefore, we conclude that the number of particles in these reactions is set by a nucleation event, which happens soon after reaching 340 °C, with a rate faster than the time resolution of our data. The first observable crystal size by PDF was 1.8 nm. Before observing crystals, the joint SAXS and PDF analysis already detected large amounts of amorphous particles. This intermediate material is a logical consequence of the high reactivity of Zr–OH moieties, produced by precursor conversion. The size of the amorphous particles was determined to be 1.5–2 nm. One could hypothesize that an amorphous particle crystallizes while maintaining its size since there is precedent for an order–disorder transition.^17^ However, there is no reason for such a process to be limited to the start of the reaction. In addition, our kinetic modeling indicates a second order nucleation event. Such a second order reaction has a strong dependence on the concentration of the intermediate, which agrees with the absence of continuous nucleation.

Nanocrystal growth happens while the number of total particles decreases over the course of the reaction, see Figure 2g. Amorphous particles are consumed by growth, which can happen by coalescence with a growing nanocrystal or by dissolution into smaller species that grow onto the nanocrystals. Distinguishing the two mechanisms is not straightforward with the data at hand. In the case of zirconium chloride, our kinetics modeling does suggest that a reversible equilibrium between intermediates and precursors is less likely than intermediates reacting with themselves or growing onto nanocrystals, thus rather supporting a coalescence mechanism, although we cannot exclude other reactive intermediates formed in low concentrations.

**Figure 5:**
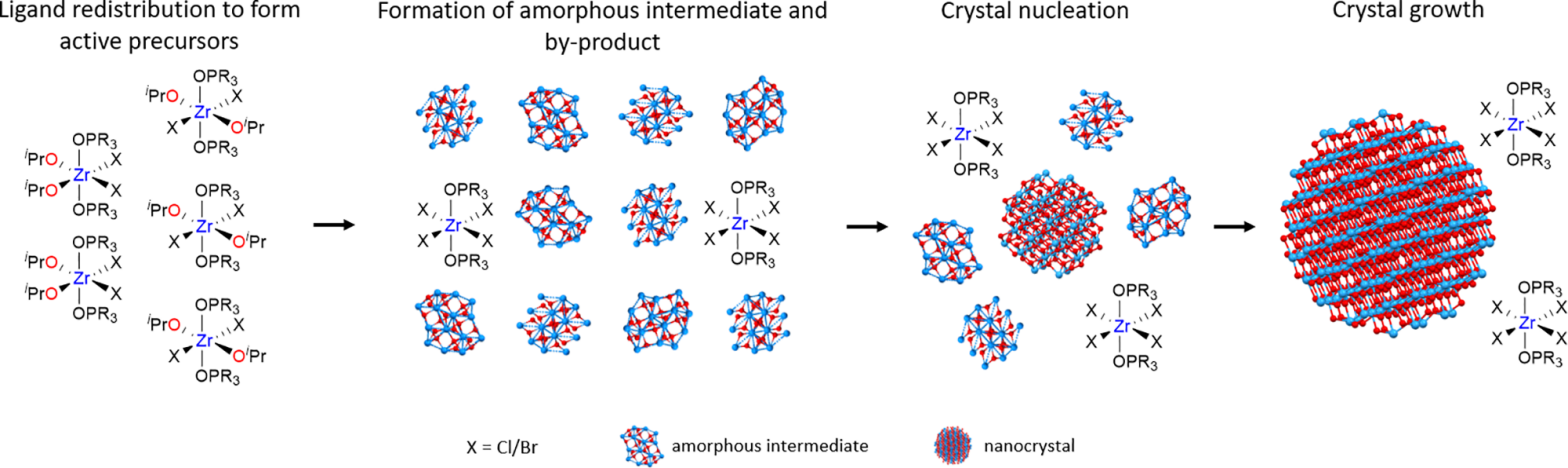
Schematic representation of the formation mechanism of zirconia nanocrystals. Precursor conversion leads to amorphous intermediates, from which crystals nucleate and grow.

A schematic representation of the proposed mechanism is given in Figure 5. After dissolution of the precursors, we obtain the mixed halide-alkoxide complexes, which decompose into amorphous particles and zirconium tetrahalide complexes. From the amorphous particles, crystals nucleate in a short nucleation event and the rest of the amorphous particles are consumed during crystal growth. We see no evidence for continuous nucleation nor ripening of the *crystalline* particles. Given that the number of nanocrystals does not change, the final nanocrystal size is set by the nucleation event, irrespective of the growth rate. A larger number of nanocrystals correlates with a smaller final size.

### Tuning the Size Using Mechanistic Insight

According to our mechanism, the number of nanocrystals (thus the size at full yield) is determined by the nucleation event, and the nucleation reaction is second order in the amorphous intermediate. The growth reaction is only first order in intermediate. We can thus favor nucleation over growth by increasing the concentration of amorphous particles at the nucleation event. A higher extent of nucleation increases the number of particles and thus decreases the size at full yield. Since all converted precursor turns into amorphous particles, our efforts were focused on controlling the precursor conversion by varying precursor structure. As shown in Figure S26a, the precursor conversion rate increases in the series of reaction mixtures:
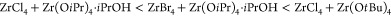
As expected, faster precursor decomposition (higher concentration of amorphous intermediate) correlates with a smaller final particle size (Figure S26b–d). The approach was further validated by modifying the ZrCl_4_ + Zr(O*i*Pr)_4_·*i*PrOH reaction. The reaction mixture was held at 300 °C for 2 h, reaching 91% precursor conversion. The solution was then quickly heated to 340 °C (Figure S27). This method was designed to again increase the number of amorphous particles at nucleation (which we assume does not happen at 300 °C). Indeed, the reaction yielded smaller particles compared to the standard reaction (S27). Finally, mixing ZrCl_4_ and ZrBr_4_ delivers an intermediate size, see Figure S28.

## Conclusion

In this work, we elucidated the nucleation and growth mechanism during the nonaqueous synthesis of ZrO_2_ nanocrystals from zirconium halide (chloride and bromide) and zirconium isopropoxide in trioctylphosphine oxide. Upon decomposition of the active precursor, a lot of amorphous nanoparticles are formed, which are subsequently converted into nanocrystals. The presence of the amorphous intermediate was evidenced by a combined analysis of X-ray total scattering, small-angle X-ray scattering, and NMR. We presented a closed mass balance and kinetic modeling. We determined that the precursor conversion rate is an order of magnitude higher than the crystallization rate. This rate imbalance is responsible for the rapid buildup of amorphous particles. Changing the halide precursor from chloride to bromide, we observed faster kinetics for both precursor conversion and crystallization, together with a smaller crystal size. We found that the nanocrystal size is generally controlled by the concentration of amorphous particles at nucleation (second order) and we showed multiple strategies for size tuning. These comprehensive results present a leap forward in our understanding of group 4 metal oxide nanocrystals and are expected to enable higher synthetic control over nanocrystal architecture.

## Experimental Section

### Materials

ZrCl_4_ (99.9%), ZrBr_4_ (99%), and Zr(O*t*Bu)_4_ (99.9%) were purchased from Strem Chemicals, and Zr(O*i*Pr)_4_·*i*PrOH (99.9%), toluene (99.5%), and acetone (99.8%) were purchased from Sigma-Aldrich and used without further purification. Deuteroform (99.8 atom %) was purchased from Cambridge Isotope Laboratories and benzene-*d*_6_ (99.5 atom %) from Apollo scientific; 10/100 mL of activated 4 Å molecular sieves were added and left to stand for 3 days in the glovebox to remove residual water. Three-millimeter high-throughput NMR tubes (0.58 mm wall thickness) were purchased from Sigma-Aldrich. Tri-*n*-octylphosphine oxide (99%) was bought from Strem chemicals and recrystallized according to Owen et al.^53^

### Instrumentation

Transmission electron microscopy (TEM) imaging was done using a JEOL JEM2800 field emission gun microscope operated at 200 kV equipped with a TVIPS XF416ES TEM camera. Nuclear magnetic resonance (NMR) measurements were recorded on Bruker spectrometers operating at a ^1^H frequency of 500.13 MHz. ^31^P spectra were acquired using inverse gated decoupling. The ^31^P spectra were processed with a line broadening of 5 Hz to reduce noise.

### ZrO_2_ Synthesis

Zirconia nanocrystals are synthesized according to our previously published procedure,^41^ which was slightly different from the original procedure of Joo et al.^39^ Typical amounts were 7.5 g recrystallized TOPO, Zr(O*i*Pr)_4_·*i*PrOH (0.387 g, 1.5 mmol), and ZrCl_4_ (0.349 g, 1.5 mmol). Synthetic variations include (i) using ZrBr_4_ (0.616 g, 1.5 mmol) instead of ZrCl_4_ and (ii) using Zr(O*t*Bu)_4_ (0.575 g, 1.5 mmol) instead of Zr(O*i*Pr)_4_·*i*PrOH. All preparations were executed in a nitrogen filled glovebox, and the actual reaction was executed on a Schlenk line operating with Ar.

### *Ex Situ* X-ray Total Scattering Experiments

Samples were prepared by the temporal sampling of reaction aliquots into 3 mm NMR tubes sealed under argon atmosphere. We also used 2 mm glass capillaries from Hilgenberg for sampling, but those were prone to breaking upon heating to 80 °C and in general did not result in higher data quality. The samples were measured either using beamline P21.1 at DESY in Hamburg, Germany or using beamline ID15A at ESRF in Grenoble, France.^54^ At ESRF, data were collected at 80 °C (using a nitrogen cryostream), in rapid acquisition mode, using a 2D Pilatus CdTe 2M detector (1679 × 1475 pixels and 172 × 172 μm pixel size) with a sample-to-detector distance of 264 mm. The incident wavelength of the X-rays was λ = 0.1441 Å (66.05 keV). Calibration of the experimental setup was performed using a Silicon standard sample. At DESY, *ex-situ* X-ray scattering data were collected at 80 °C in a home-built aluminum heating block (see in situ experiments) in rapid acquisition mode, using a 2D Varex 4343RF amorphous silicon detector (2880 × 2880 pixels and 150 × 150 μm pixel size) with a sample to detector distance of 800 mm. During the measurement, the sample stage was placed in a helium filled chamber to avoid air scattering. The incident wavelength of the X-rays was λ = 0.1220 Å (101.62 keV). Calibration of the experimental setup was performed using a Ni standard sample.

### *In Situ* X-ray Total Scattering Experiments

Samples were prepared in a nitrogen-filled glovebox by mixing the precursors (see ZrO_2_ synthesis) in molten tri-*n*-octylphosphine oxide. The mixture was then transferred to a 3 mm NMR tube and sealed. The samples were measured using beamline P21.1 at DESY in Hamburg, Germany. A home-built aluminum block with an NMR tube holder and cartridge heaters was used to establish the reaction environment. The temperature was first ramped to 340 °C followed by a plateau for 2 h with Lakeshore 336 cryogenic temperature controller. The sample stage was placed in a helium-filled chamber to avoid air scattering. The data were continuously collected in rapid acquisition mode using a 2D Varex 4343RF amorphous silicon detector (2880 × 2880 pixels and 150 × 150 μm pixel size) with a sample-to-detector distance of 800 mm and an exposure time of 2 s. The incident wavelength of the X-rays was λ = 0.1220 Å (101.62 keV). Calibration of the experimental setup was performed using a Ni standard sample.

### Analysis of X-ray Total Scattering Data

Raw 2D data were corrected for geometrical effects and polarization, then azimuthally integrated to produce 1D scattering intensities versus the magnitude of the momentum transfer *Q* (where *Q* = 4π sin θ/λ for elastic scattering) using the program Fit2D. The program xPDFsuite with PDFgetX3 was used to perform the background subtraction, further corrections, the normalization to obtain the reduced total scattering structure function *F*(*Q*), and Fourier transformation to obtain the pair distribution function (PDF), *G*(*r*).^55,56^ The refinement for each data point was carried out using Diffpy-CMI with a dual-phase fit.^57^

### Small-Angle X-ray Scattering Experiments

The SAXS experiments were performed on the SWING beamline at SOLEIL synchrotron (Saint Aubin, France). The sample to SAXS detector distances was 0.5 m (for the ZrBr_4_ synthesis) and 3 m (for the ZrCl_4_ synthesis). Aliquots collected at different stages of the reactions (during the heating ramp at 300 °C, 320 °C, 340 °C and after 1, 3, 6, 9, 12, 15, 20, 25, 30, 60, 90, and 120 min at 340 °C). The samples were measured in a flow-through setup which enables the scattering patterns of the empty capillary, the solvent (toluene), and the solutions to be measured at exactly the same spot in the capillary. This allows the same background signals to be subtracted and to obtain the pattern on an absolute scale. Each sample was measured 10 times and the signals were then averaged. The 2D SAXS images were radially averaged using beamline-specific procedures. Then, the capillary signals were subtracted. The signal of the toluene solvent was used to obtain an absolute scale. From the isothermal compressibility of toluene, the intensity at high *q* was 0.0026 mm^–1^. This was used to determine a multiplying factor that was applied to all the samples. The solvent signal was then subtracted to obtain all samples in absolute intensity (Figure 2a,b). Details of the fitting are located in Supporting Information.

## Data Availability

The data that support the findings of this study are openly available in Zenodo at https://doi.org/10.5281/zenodo.7829202. A preprint of this manuscript is available on ChemRxiv: Pokratath, R.; Lermusiaux, L.; Checchia, S.; Mathew, J. P.; Cooper, S. R.; Mathiesen, J. K.; Landaburu, G.; Banerjee, S.; Tao, S.; Reichholf, N.; Billinge, S. J. L.; Abécassis, B.; Jensen, K. M. Ø.; De Roo, J. An amorphous phase precedes crystallization; unraveling the colloidal synthesis of zirconium oxide nanocrystals. *ChemRxiv*, February 10, 2023. DOI: 10.26434/chemrxiv-2023-9bnjv-v2 (accessed on April 6, 2023).

## Supporting Information Available

Details of SAXS data processing, PDF refinements, NMR analysis of reaction aliquots, mechanism fitting using COPASI, TEM and NMR data for size and concentration tuning (PDF)
