# Supplementary material for: An Amorphous Phase Precedes Crystallization: Unraveling the Colloidal Synthesis of Zirconium Oxide Nanocrystals
Source: ACS Nano. 2023 Apr 24;17(9):8796–806. doi: 10.1021/acsnano.3c02149 (PMC10173684; doi:10.1021/acsnano.3c02149)
Supplement: Supplementary file 1 — nn3c02149_si_001.pdf [file nn3c02149_si_001.pdf]

Supporting Information: An amorphous phase precedes crystallization: unraveling the colloidal synthesis of zirconium oxide nanocrystals

Rohan Pokratath,[†] Laurent Lermusiaux,[‡] Stefano Checchia,[¶] Jikson Pulparayil Mathew,[†] Susan Rudd Cooper,[§] Jette Katja Mathiesen,[§,‖] Guillaume Landaburu,[‡] Soham Banerjee,[⊥] Songsheng Tao,[#] Nico Reichholf,[†] Simon J. L. Billinge,[#] Benjamin Abécassis,[‡] Kirsten M. Ø. Jensen,[§] and Jonathan De Roo[*,†]

†*Department of Chemistry, University of Basel, Mattenstrasse 24a, 4058 Basel, Switzerland*

‡*ENSL, CNRS, Laboratoire de Chimie 745 UMR 5182, 69364 Lyon, France*

¶*ESRF Synchrotron, ID15A Beamline, 71 Avenue des Martyrs, CS40220, 38043 Grenoble, France*

§*Department of Chemistry, University of Copenhagen, Universitetsparken 5, 2100 Copenhagen Ø, Denmark*

‖*Department of Physics, Technical University of Denmark, Fysikvej Bldg. 312, 2800 Kgs. Lyngby, Denmark*

⊥*Deutsches Elektronen-Synchrotron DESY, Notkestraße 85, 22607 Hamburg, Germany*

#*Applied Physics and Applied Mathematics Department, Columbia University, New York, 10027 NY, USA*

E-mail: Jonathan.DeRoo@unibas.ch

SAXS data fitting

The normalized SAXS patterns are used to determine the particle size and concentration by fitting the experimental intensity. As mentioned in the manuscript, we use as a fitting function the sum of the scattering cross section of a distribution of polydisperse spheres and the experimental signal obtained at 300 °C for the $ZrCl_4$ reaction mixture multiplied by a fitting parameters (F) set between 0 and 1:

$$I_{fit} = I^{theoretical}_{Polydisperse\ spheres} + F * I^{experimental}_{\mathrm{ZrCl_4}\ at\ 300°\mathrm{C}}$$

The theoretical scattering cross section of distribution of spheres of homogeneous electron density dispersed in a solvent is given by:

$$I^{theoretical}_{Polydispersespheres} = n(\Delta\rho)^2 \int_0^\infty V(R)^2 D(R) P(q,R) dR$$

where n is the particle concentration, $\Delta\rho$ is the difference in scattering length density between the particles and the solvent (the contrast), V(R) is the volume of a sphere of radius R, D(R) is the radius distribution, and P(q,R) is the form factor of a sphere. Here, we use a Schultz distribution with

$$D(R) = (\frac{R}{R_0})(Z+1)^{(Z+1)} \frac{e^{-(Z+1)\frac{R}{R_0}}}{\Gamma(Z+1)}$$

Z is related to the polydispersity of the distribution as the polydispersity

$$P(\%) = \frac{100}{\sqrt{Z+1}}$$

The fit function can be written as

$$I_{fit} = I_{Schultz}(n, R_0, Z) + F * I^{experimental}_{\mathrm{ZrCl_4}\ at\ 300^oC}$$

with 4 fitting parameters (R, Z, n and F). In order to provide an estimation of the error

bars for the desired parameters (R and n), we performed fits with a fixed value of Z. For each SAXS pattern, Z was varied using increments of 1 from 1 to 500 and we evaluated the quality of the fit using χ^2 within this Z range (Figure S1a). We measure the χ^2 value for which the fit is minimal (χ^2_{min}) and extract the two Z values which correspond to an increase of χ^2 by 10 %: $\chi^2 = \chi^2_{min} * 1.1$. We then use these two Z values, Z_- and Z_+, to extract the corresponding R_{min} and R_{max}, n_{min} and n_{max}, which serve as the limits of the error-bars (Figure S1b-c).

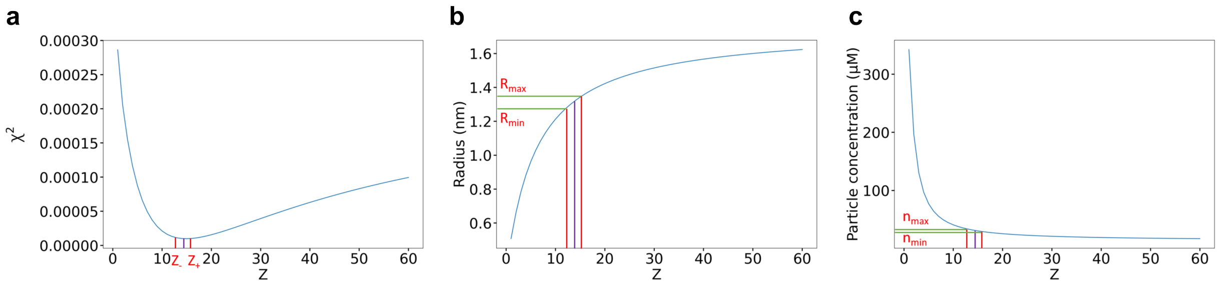

Figure S1: Determination of the error-bar in SAXS data. (**a**) Example, for one sample, of the estimation of the quality of the fit, using χ^2, when varying the parameter Z, Z_- and Z_+ correspond to Z when χ^2 is 10 % larger than its minimum. Variation of the radius (**b**) and concentration (**c**) given by the fit when Z is varied.

To account for the intensity of the SAXS signal resulting from the precursors ($I_{precursors}$), we used in our fits the experimental signal of the $ZrCl_4$ reaction at 300 °C ($F * I^{experimental}_{ZrCl_4\ at\ 300^oC}$), even for the $ZrBr_4$ reaction, which may be surprising. First, all the fits were also performed using the SAXS signals of the respective precursors measured at room temperature. Although the fits were visually not as good, the final results were very similar (Figure S2). Second, for the $ZrCl_4$ synthesis at 300 °C, NMR shows that there is no conversion of the precursors at this stage, confirming this signal can be appropriately used in the fitting. However, this was not the case for the $ZrBr_4$ synthesis which has a faster kinetics, explaining why the $ZrBr_4$ reaction at 300 °C could not be used in the fitting.

The synthesis yield is calculated by dividing the volume of synthesized particles at a given

point by the total volume of particles possibly synthesized (estimated from the quantities of chemicals used). The estimation of the yield by SAXS is obtained by multiplying the number of synthesized particles by the average particle volume $< V >$ with:

$$< V >= \frac{4}{3}\pi \int_0^\infty f(R)R^3 dR$$

and with f(R) given by the Schultz distribution:[S1]

$$f(R) = \left[\frac{Z+1}{< R >}\right]^{Z+1} R^Z exp\left[-\left(\frac{Z+1}{< R >}R\right)\right]\frac{1}{\Gamma(Z+1)}$$

Here, Z is related to the width of the distribution and $< R >$ the mean sphere radius, both determined by the fits.

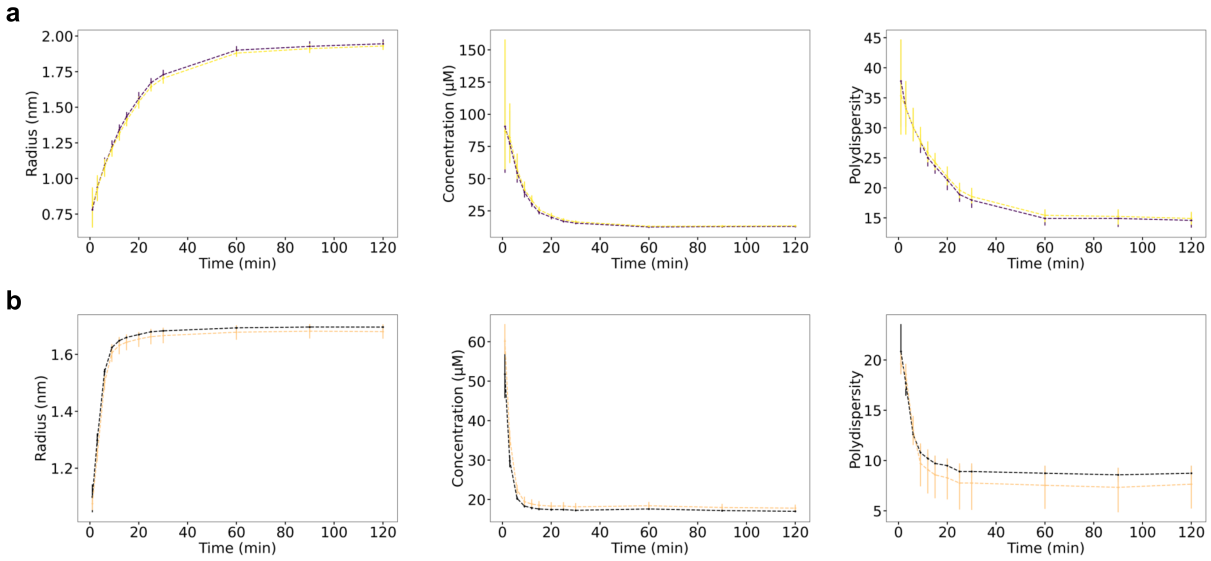

Figure S2: Effect of the precursor signal used in the fitting of the *ex situ* SAXS measurements. Comparison of the time evolution of the particle size, concentration, and polydispersity for the (**a**) $ZrCl_4$: $Zr(O\textit{i}Pr)_4 \cdot \textit{i}PrOH$ and (**b**) $ZrBr_4$: $Zr(O\textit{i}Pr)_4 \cdot \textit{i}PrOH$ syntheses. The yellow lines were obtained using in the fits the signal of the corresponding precursors measured at room temperature. The dark lines were obtained using the signal from the $ZrCl_4$ reaction at 300 °C.

PDF and NMR analysis

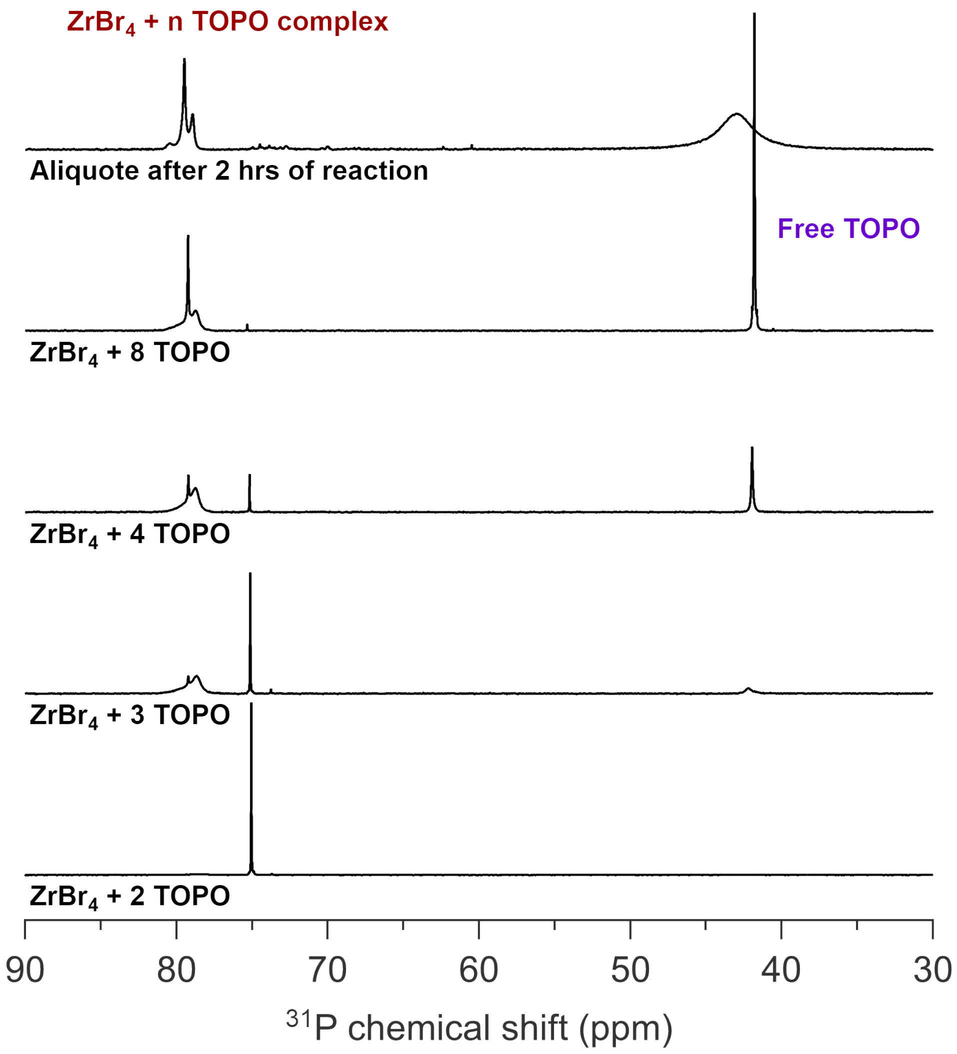

Figure S3: ^{31}P NMR spectra in C_6D_6 for reaction aliquot after 2 hours for 1:1 mixture of $ZrBr_4$: $Zr(OiPr)_4 \cdot iPrOH$ showing the formation of $ZrBr_4$ + n TOPO complex as a byproduct. The NMR shift for the complexes is independently verified by mixing $ZrBr_4$ with different equivalents of TOPO. While two TOPO equivalents seem to cleanly yield the expected $ZrBr_4 \cdot 2\,$TOPO complex, higher TOPO equivalents result in an unknown complex.

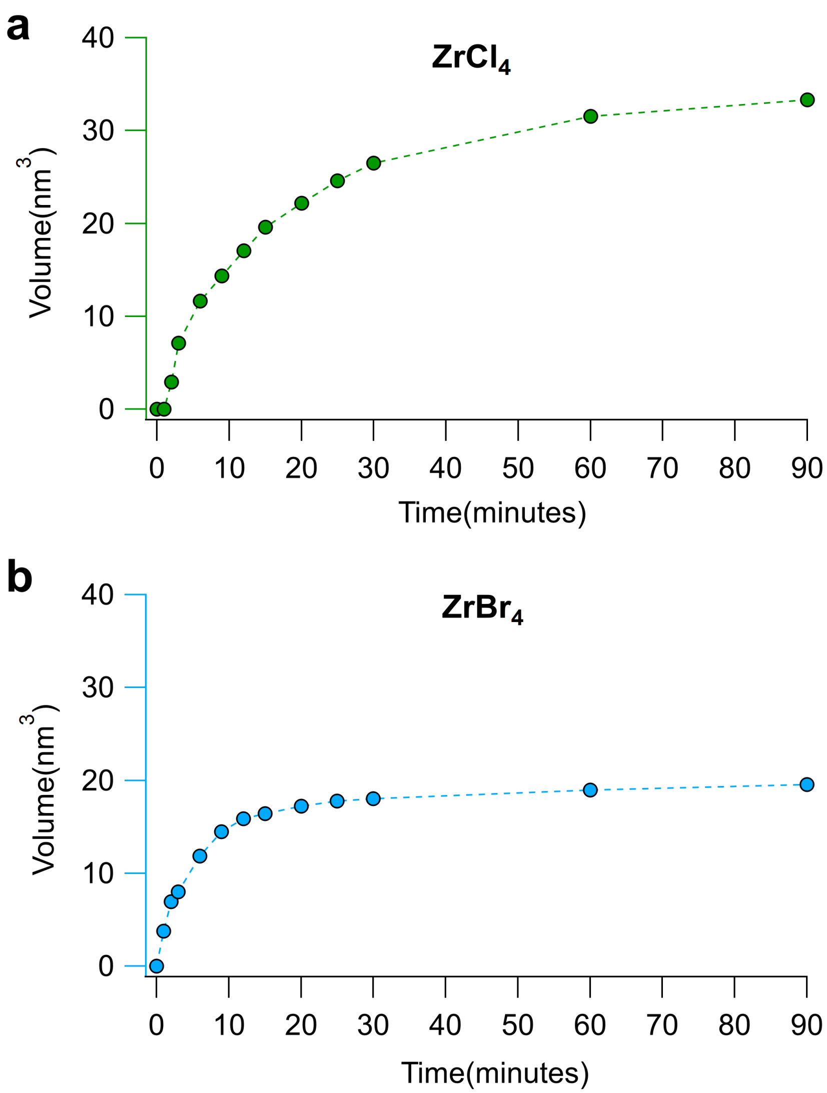

Figure S4: Comparison of volumetric changes during crystal growth for the reaction with (**a**) 1:1 mixture of $ZrCl_4$: $Zr(O\textit{i}Pr)_4 \cdot \textit{i}PrOH$. and (**b**) 1:1 mixture of $ZrBr_4$: $Zr(O\textit{i}Pr)_4 \cdot \textit{i}PrOH$.

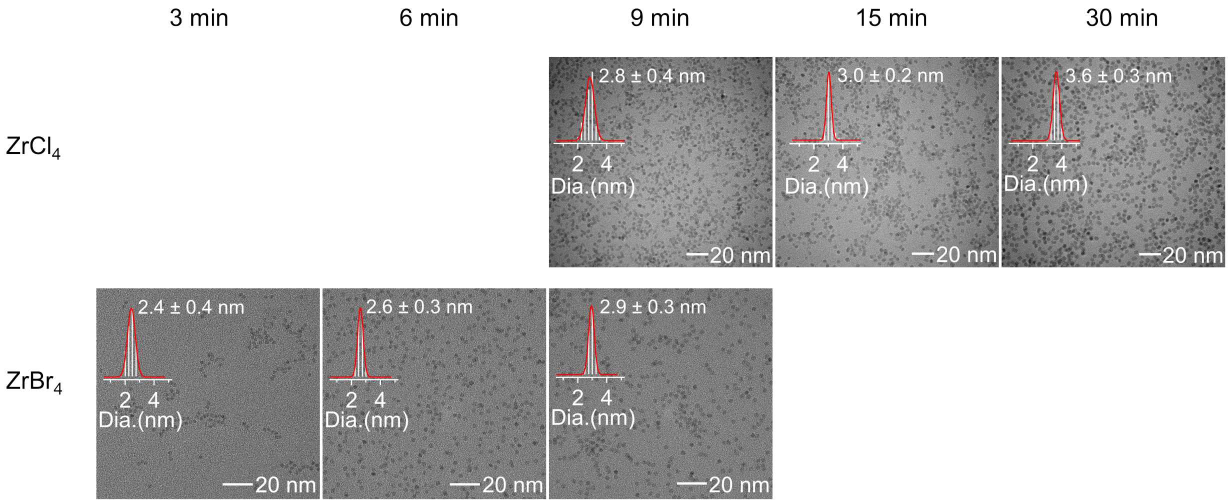

Figure S5: TEM image and histogram of particles formed from 1:1 reaction mixture of $ZrCl_4$: $Zr(O\textit{i}Pr)_4 \cdot \textit{i}PrOH$ (after 9, 15, and 30 minutes) and from 1:1 reaction mixture of $ZrBr_4$: $Zr(O\textit{i}Pr)_4 \cdot \textit{i}PrOH$ (after 3, 6, and 9 minutes).

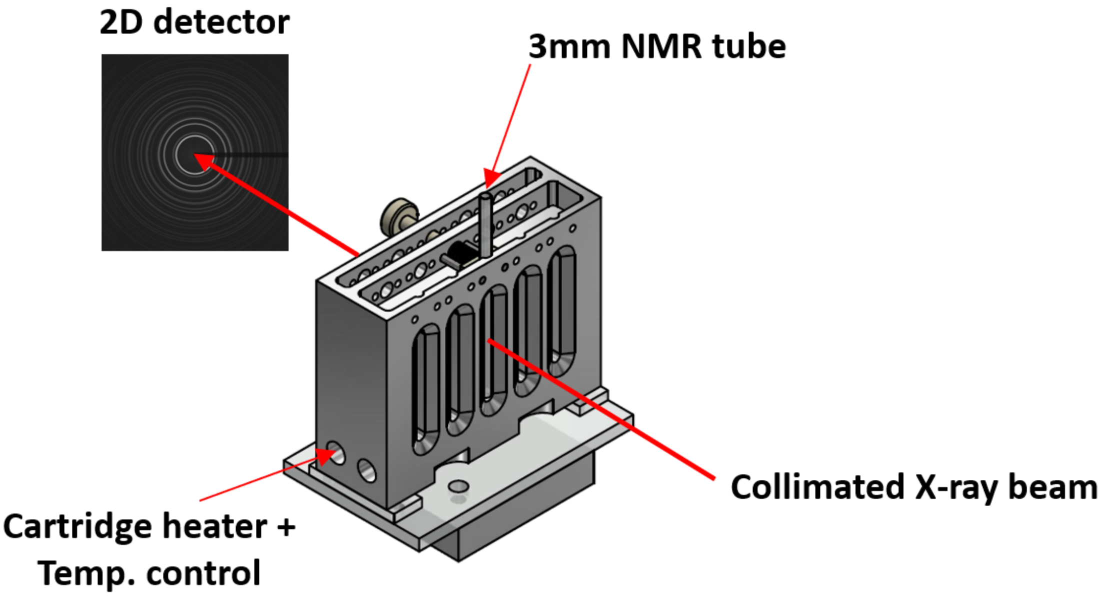

Figure S6: Custom made experimental setup for *in situ* PDF measurement. The reaction mixture is inserted in a 3 mm NMR tube and placed inside the setup. Cartridge heaters are used to heat the block.

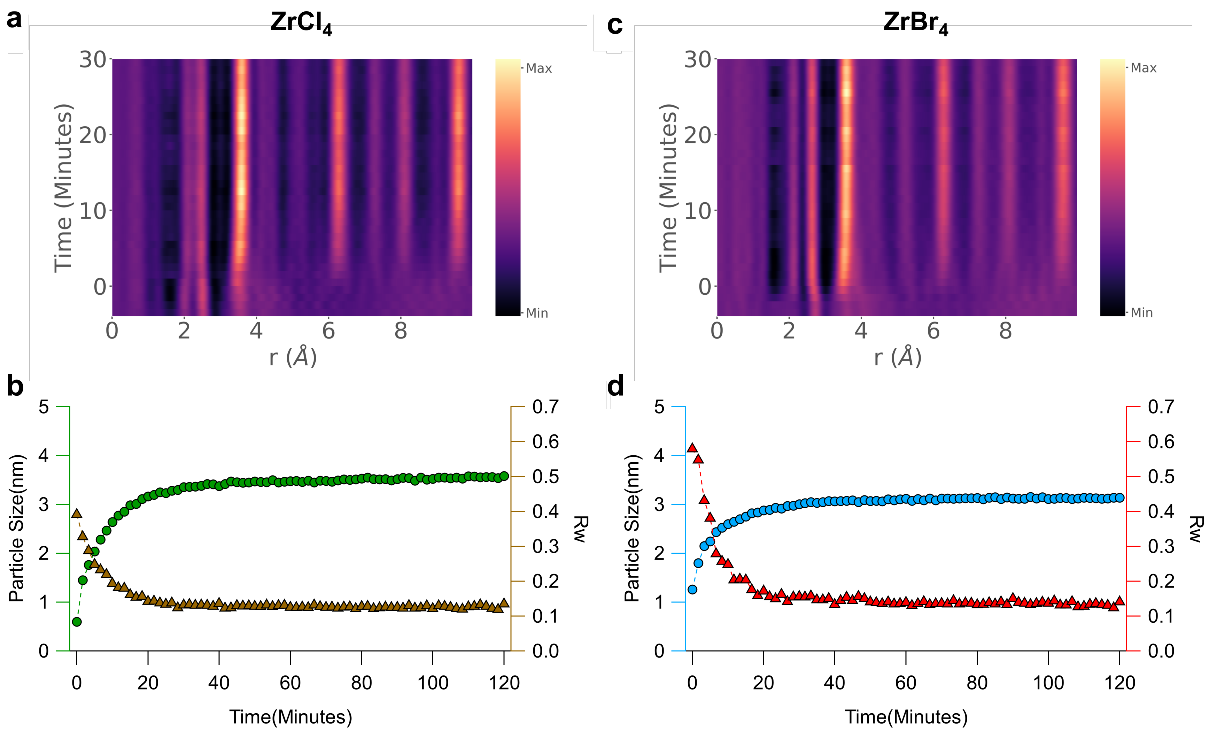

Figure S7: *In situ* PDF intensity plot and refined parameters. *In situ* PDF data and refined parameters (size and *Rw*) for 1:1 mixture of (**a-b**) $ZrCl_4$: $Zr(O\textit{i}Pr)_4 \cdot \textit{i}PrOH$ and 1:1 mixture of (**c-d**) $ZrBr_4$: $Zr(O\textit{i}Pr)_4 \cdot \textit{i}PrOH$.

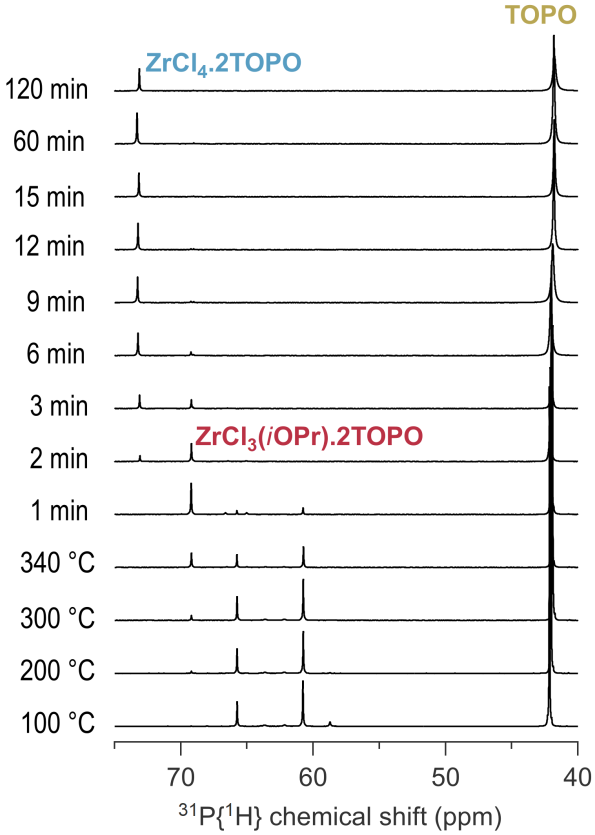

Figure S8: ^{31}P NMR of aliquotes from 1:1 reaction mixture of $ZrCl_4$: $Zr(O\textit{i}Pr)_4 \cdot \textit{i}PrOH$.

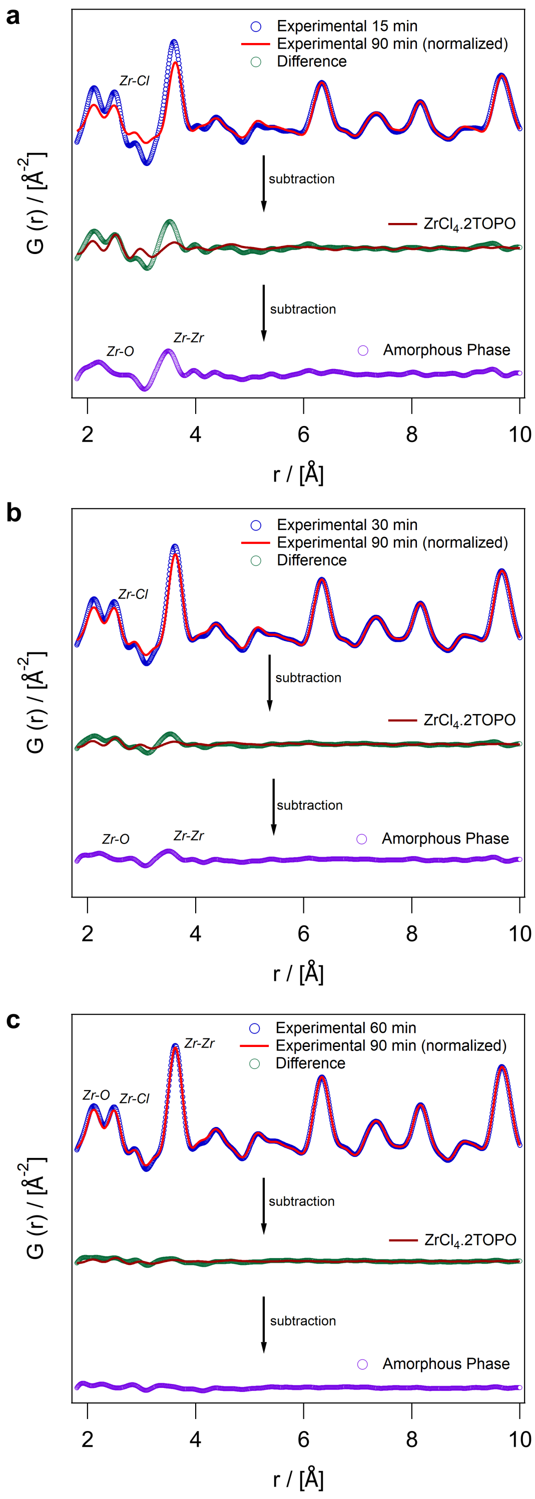

Figure S9: Extraction of the PDF of amorphous phase after 15 (**a**), 30 (**b**), and 60 (**c**) minutes for $ZrCl_4$: $Zr(O\textit{i}Pr)_4 \cdot \textit{i}PrOH$ reaction.

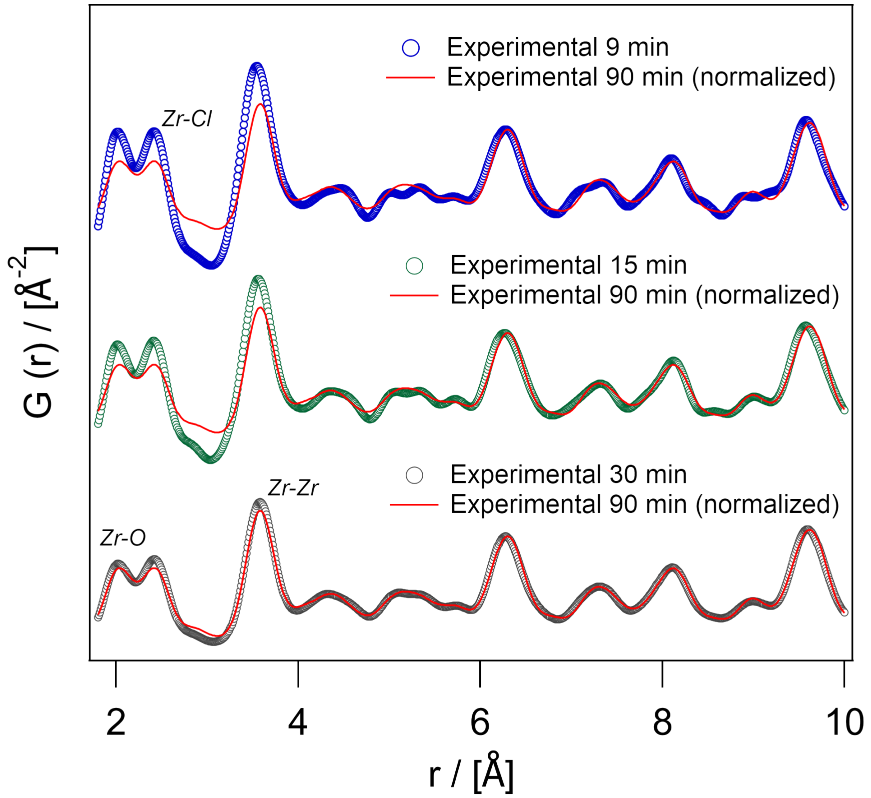

Figure S10: Changes in the contribution of amorphous intermediates captured *in situ* after 9, 15, and 30 minutes of $ZrCl_4$: $Zr(O\textit{i}Pr)_4 \cdot \textit{i}PrOH$ reaction. Each data point is normalized with reaction crude product (data point after 90 minutes) by scaling the peak intensity at 6.3 Å. The difference in the PDFs narrows with time, indicating the disappearance of amorphous intermediate. Unlike the *ex situ* data, the complete extraction of the amorphous PDF is not possible due to the unavailability of the experimental PDF of $ZrCl_4 \cdot 2\,TOPO$ under similar conditions.

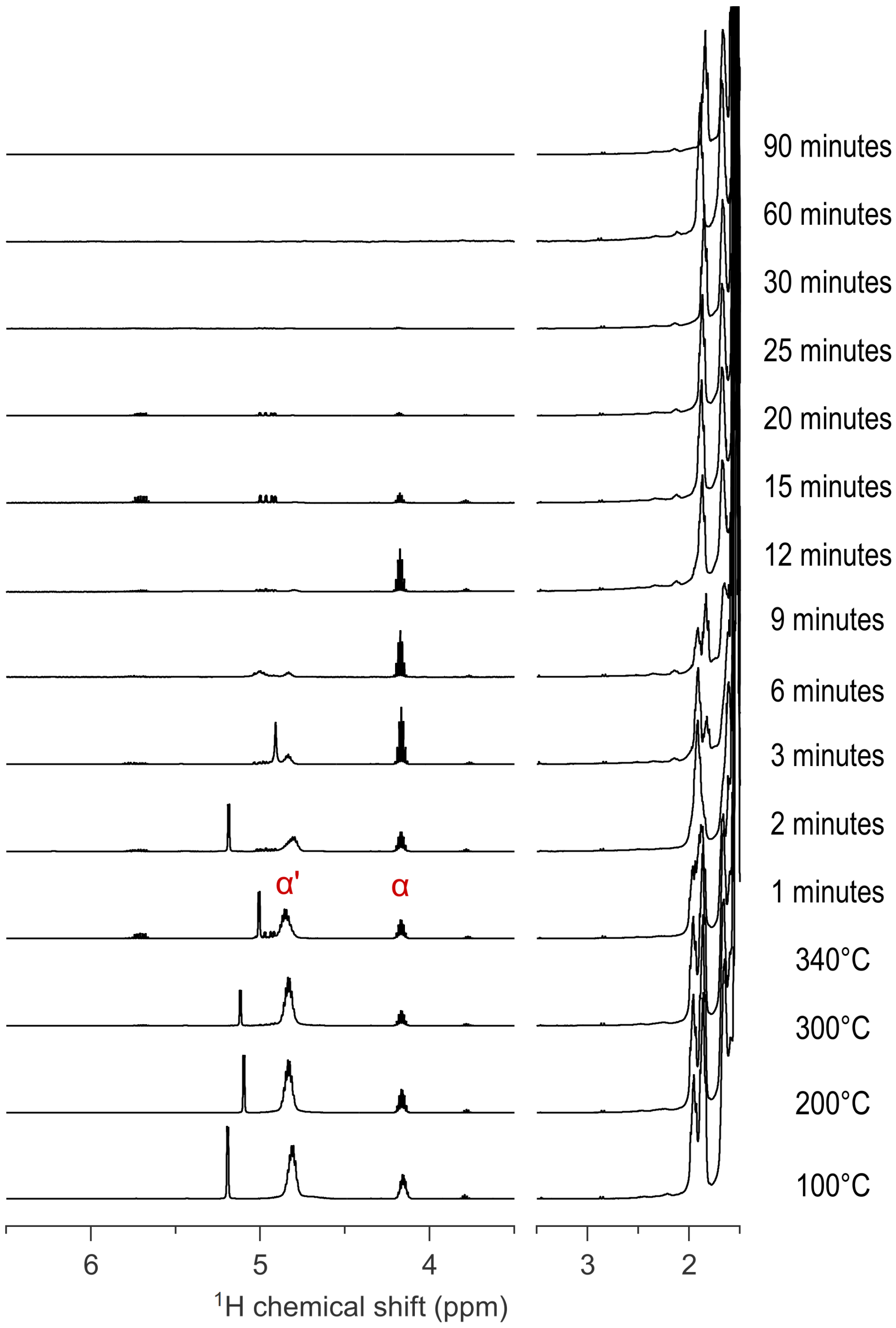

Figure S11: ^{1}H NMR spectra of the reaction $ZrCl_4$: $Zr(O\textit{i}Pr)_4 \cdot \textit{i}PrOH$ in C_6D_6. Aliquots were taken at different temperatures during the ramp and at different times at the final reaction temperature of 340 °C. The bound (α') and unbound (α) propoxide groups are indicated. The integral of α' was used for quantification.

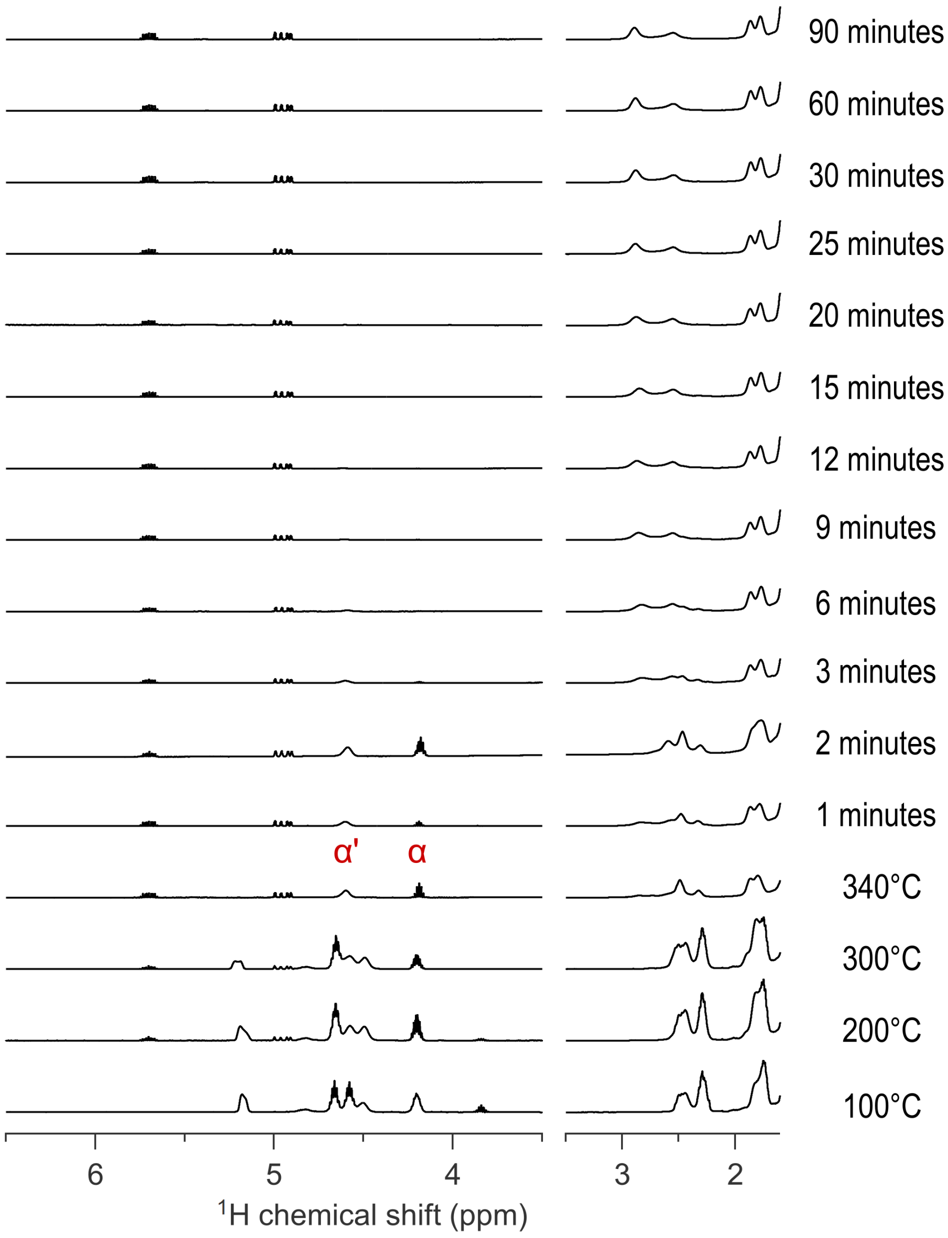

Figure S12: ^{1}H NMR spectra of the reaction $ZrBr_4$: $Zr(O\textit{i}Pr)_4 \cdot \textit{i}PrOH$ in C_6D_6. Aliquots were taken at different temperatures during the ramp and at different times at the final reaction temperature of 340 °C. The bound (α') and unbound (α) propoxide groups are indicated. The integral of α' was used for quantification.

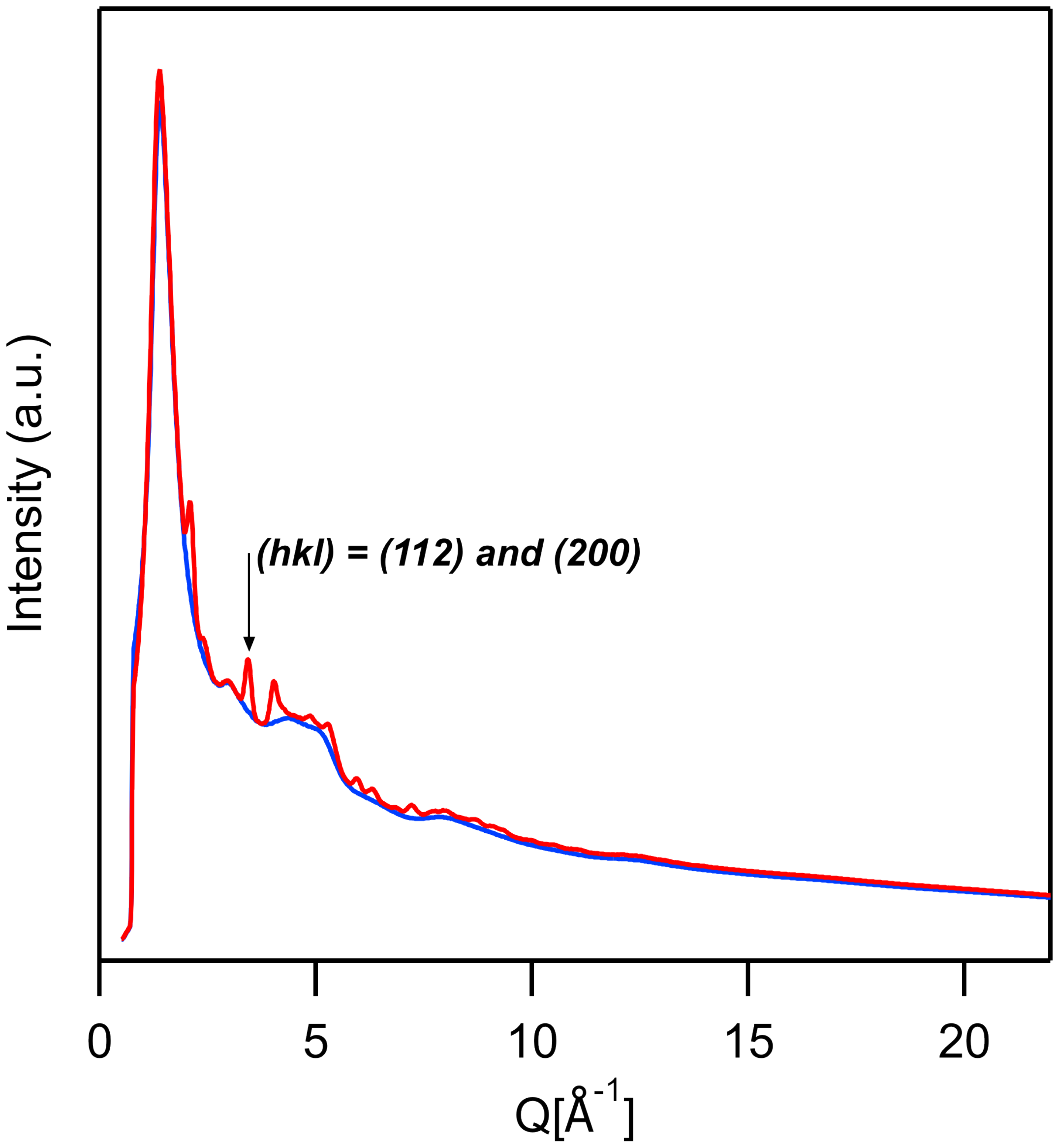

Figure S13: Calculation of area under the Bragg peak by subtracting the background (blue) from the reciprocal data (red). The Miller indices corresponding to the Bragg peak are indicated considering the crystal structure is tetragonal zirconia.

Table S1: Refined parameters after fitting aliquots from 1:1 reaction mixture of $ZrCl_4$: $Zr(OiPr)_4 \cdot iPrOH$.

**Time[min]**	**scale**	**psize[Å]**	**a[Å]**	**b [Å]**	**Uiso Zr[Å^2]**	**Uiso O[Å^2]**	***Rw***	**Amplitude**	**wasyn**	λ	ϕ	θ	**wsig**
**0**	-	-	-	-	-	-	-	-	-	-	-	-	-
**1**	-	-	-	-	-	-	-	-	-	-	-	-	-
**2**	0.27	17.70	3.68	4.98	0.065	0.075	0.72	-	-	-	-	-	-
**3**	0.21	23.85	3.64	5.12	0.028	0.068	0.53	-	-	-	-	-	-
**6**	0.14	28.13	3.66	5.09	0.016	0.077	0.34	-	-	-	-	-	-
**9**	0.12	30.14	3.63	5.21	0.013	0.052	0.16	-	-	-	-	-	-
**12**	0.18	31.95	3.63	5.21	0.012	0.056	0.13	-	-	-	-	-	-
**15**	0.22	33.46	3.63	5.22	0.011	0.052	0.12	-	-	-	-	-	-
**20**	0.28	34.86	3.63	5.22	0.011	0.054	0.10	-	-	-	-	-	-
**25**	0.34	36.09	3.63	5.22	0.010	0.054	0.09	-	-	-	-	-	-
**30**	0.41	36.99	3.63	5.23	0.009	0.052	0.09	-	-	-	-	-	-
**60**	0.56	39.19	3.63	5.23	0.009	0.051	0.08	-	-	-	-	-	-
**90**	0.54	39.92	3.63	5.23	0.009	0.052	0.08	-	-	-	-	-	-

Table S2: Refined parameters after fitting aliquots from 1:1 reaction mixture of $ZrBr_4$: $Zr(OiPr)_4 \cdot iPrOH$.

**Time[min]**	**scale**	**psize[Å]**	**a[Å]**	**b [Å]**	**Uiso Zr[Å^2]**	**Uiso O[Å^2]**	***Rw***	**Amplitude**	**wasyn**	λ	ϕ	θ	**wsig**
**0**	-	-	-	-	-	-	-	-	-	-	-	-	-
**1**	0.10	19.28	3.64	5.11	0.018	0.038	0.30	-0.303	1.615	4.837	0.050	5.154	1.899
**2**	0.12	23.65	3.62	5.20	0.014	0.051	0.25	-0.319	1.31	4.92	0.016	4.83	2.37
**3**	0.12	24.82	3.62	5.20	0.014	0.050	0.21	-0.455	1.876	4.804	0.064	0.557	2.49
**6**	0.12	28.29	3.62	5.21	0.011	0.057	0.15	-0.715	2.433	4.702	0.135	-7.468	2.498
**9**	0.17	30.02	3.62	5.21	0.011	0.061	0.12	-3.744	2.357	4.70	1.193	-20.049	3.462
**12**	0.18	31.17	3.62	5.21	0.010	0.058	0.12	-0.217	2.497	4.695	1.150	-14.485	2.873
**15**	0.14	31.55	3.62	5.21	0.010	0.057	0.12	-14.062	2.787	4.619	0.166	-30.887	3.228
**20**	0.14	32.04	3.62	5.21	0.010	0.057	0.11	-13.528	2.760	4.639	0.162	-30.346	3.234
**25**	0.15	32.38	3.62	5.21	0.010	0.054	0.11	-12.297	2.804	4.638	0.154	-30.286	3.241
**30**	0.15	32.54	3.62	5.21	0.010	0.057	0.11	-11.654	2.716	4.648	0.146	-28.363	3.202
**60**	0.16	33.09	3.62	5.21	0.010	0.055	0.11	-10.797	2.733	4.685	0.137	-28.056	3.186
**90**	0.19	33.42	3.62	5.21	0.010	0.053	0.11	-8.379	2.681	4.699	0.119	-25.516	3.141

Mechanism fitting

This experimental data in Fig. 4b-c was used for fitting in COPASI 4.35.[S2] When preparing the data for the program, an x-shift of 0.7 min was given for $ZrCl_4$ and 1 min for $ZrBr_4$ to take the ramping into consideration. One cannot start at time = 0 with a precursor conversion that has already progressed by 80 %.

Reaction with $ZrCl_4$

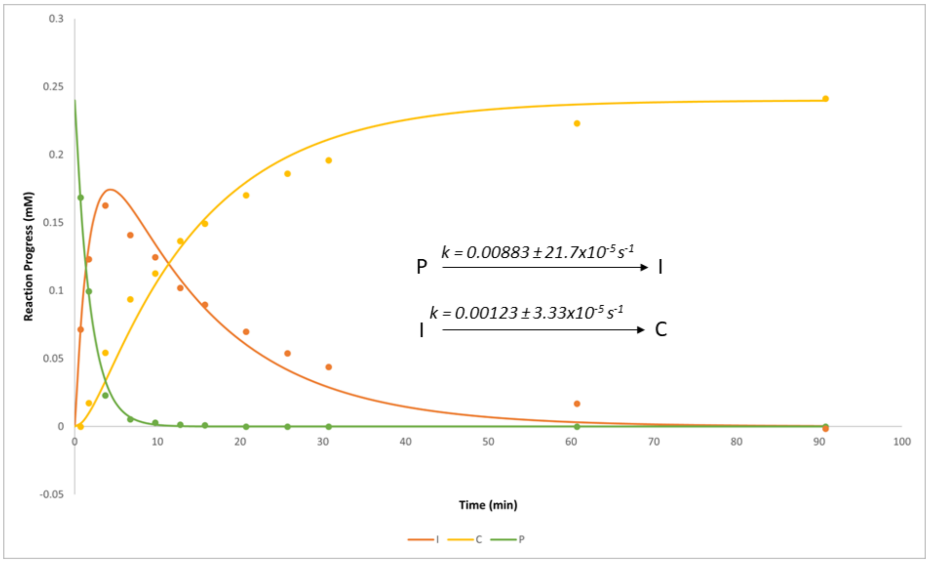

Figure S14: Fitting for $ZrCl_4$: $Zr(O\textit{i}Pr)_4 \cdot \textit{i}PrOH$ reaction data with mechanism 3. The root mean square (RMS) value for the fit is 0.00498.

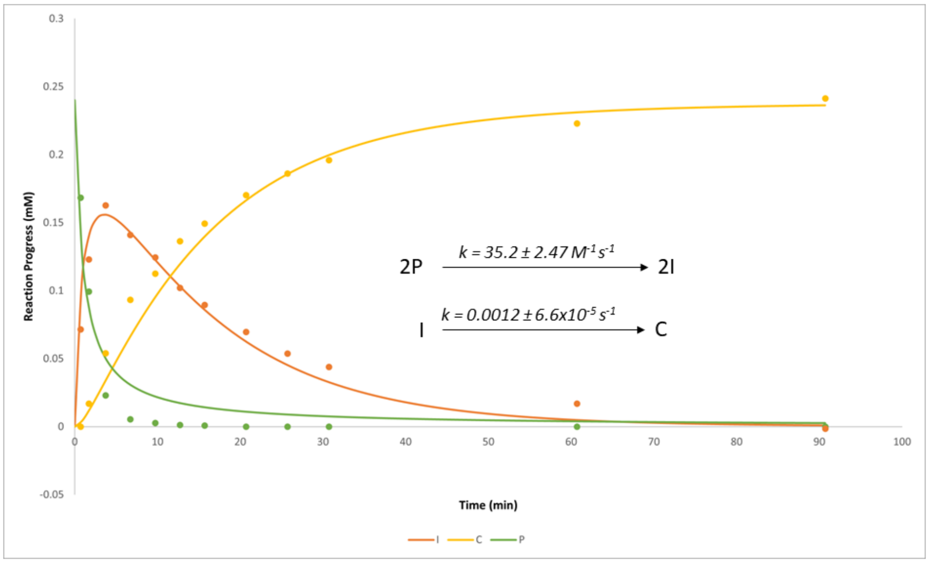

Figure S15: Fitting for $ZrCl_4$: $Zr(O\textit{i}Pr)_4 \cdot \textit{i}PrOH$ reaction data with mechanism 3 with second-order kinetics in step 1. The root mean square (RMS) value for the fit is 0.01076.

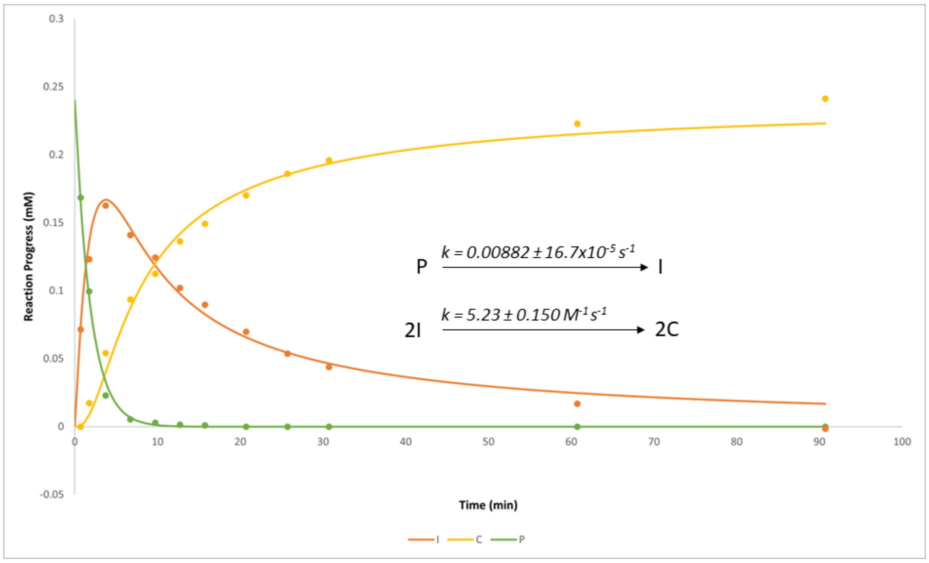

Figure S16: Fitting for $ZrCl_4$: $Zr(O\textit{i}Pr)_4 \cdot \textit{i}PrOH$ reaction data with mechanism 3 with second-order kinetics in step 2. The root mean square (RMS) value for the fit is 0.00374.

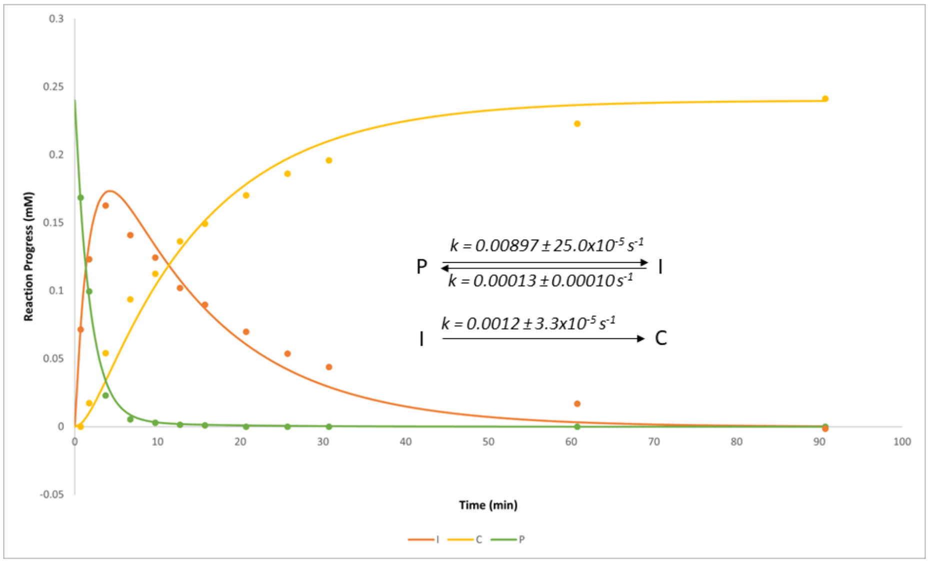

Figure S17: Fitting for $ZrCl_4$: $Zr(O\textit{i}Pr)_4 \cdot \textit{i}PrOH$ reaction data with mechanism 3 with reversible kinetics in step 1. The root mean square (RMS) value for the fit is 0.00492.

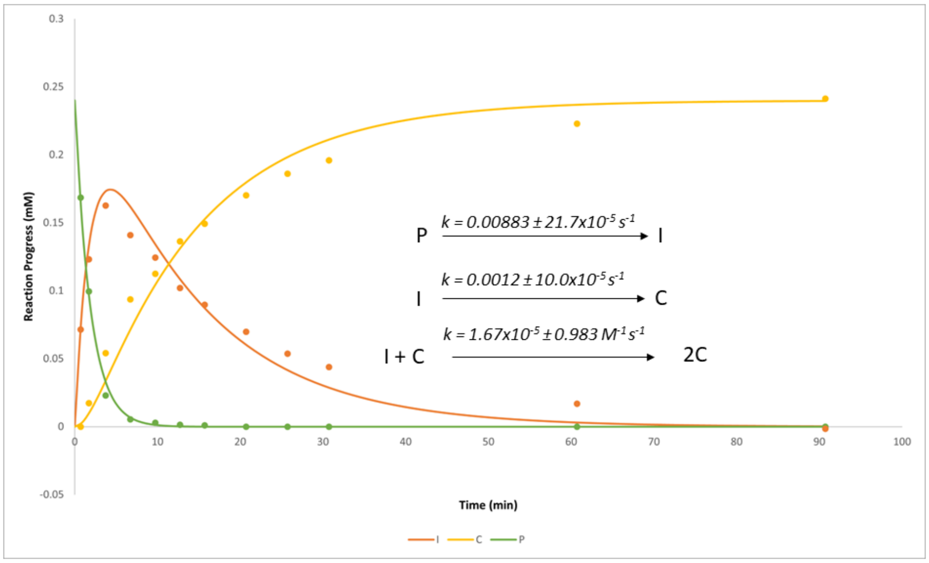

Figure S18: Fitting for $ZrCl_4$: $Zr(O\textit{i}Pr)_4 \cdot \textit{i}PrOH$ reaction data with mechanism 4 with first-order kinetics in step 2. The root mean square (RMS) value for the fit is 0.00498.

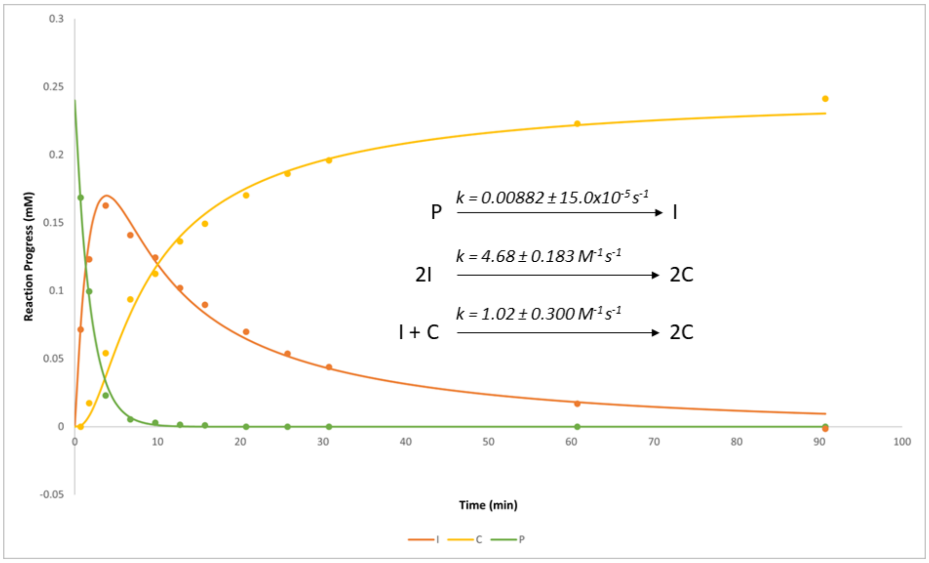

Figure S19: Fitting for $ZrCl_4$: $Zr(O\textit{i}Pr)_4 \cdot \textit{i}PrOH$ reaction data with mechanism 4. The root mean square (RMS) value for the fit is 0.00337.

Reaction with $ZrBr_4$

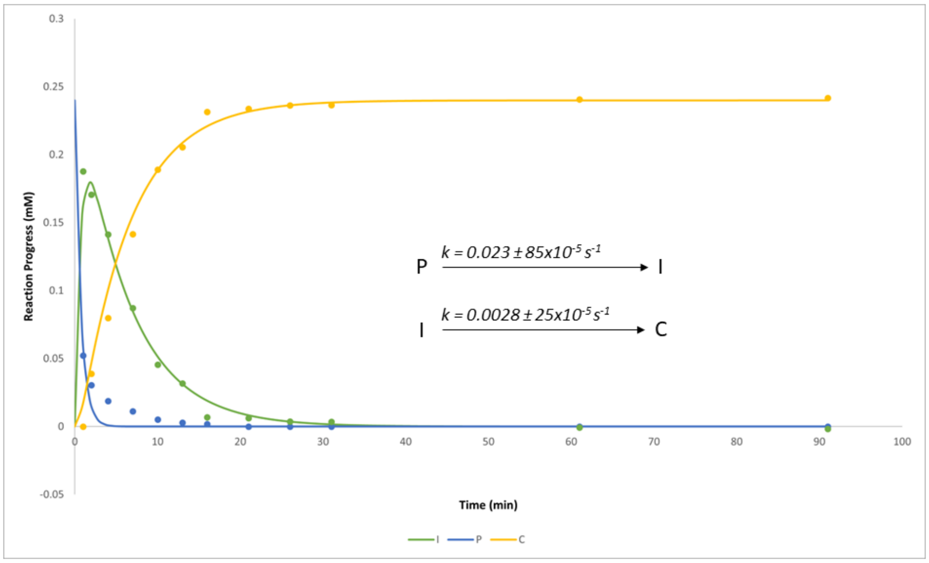

Figure S20: Fitting for $ZrBr_4$: $Zr(O\textit{i}Pr)_4 \cdot \textit{i}PrOH$ reaction data with mechanism 3. The root mean square (RMS) value for the fit is 0.00480.

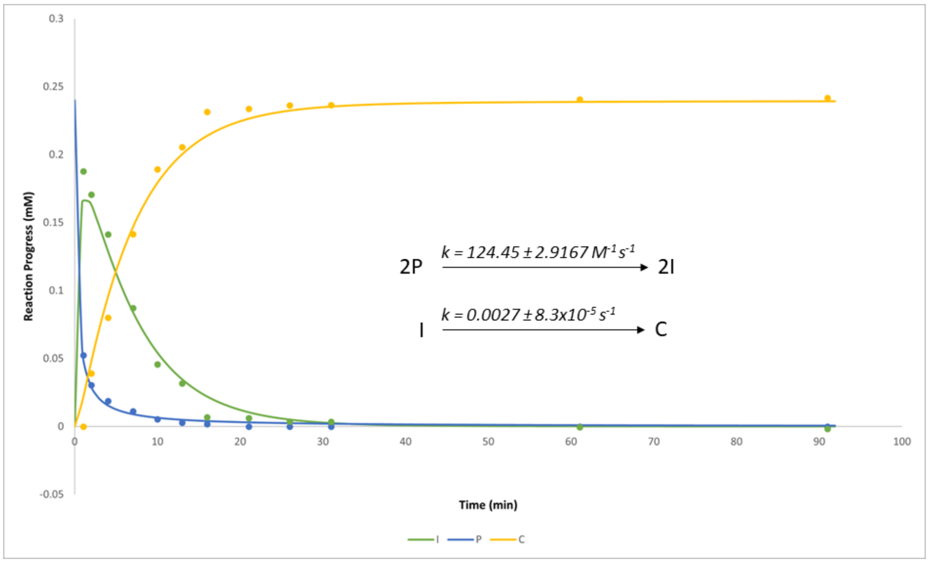

Figure S21: Fitting for $ZrBr_4$: $Zr(O\textit{i}Pr)_4 \cdot \textit{i}PrOH$ reaction data with mechanism 3 with second-order kinetics in step 1. The root mean square (RMS) value for the fit is 0.00171.

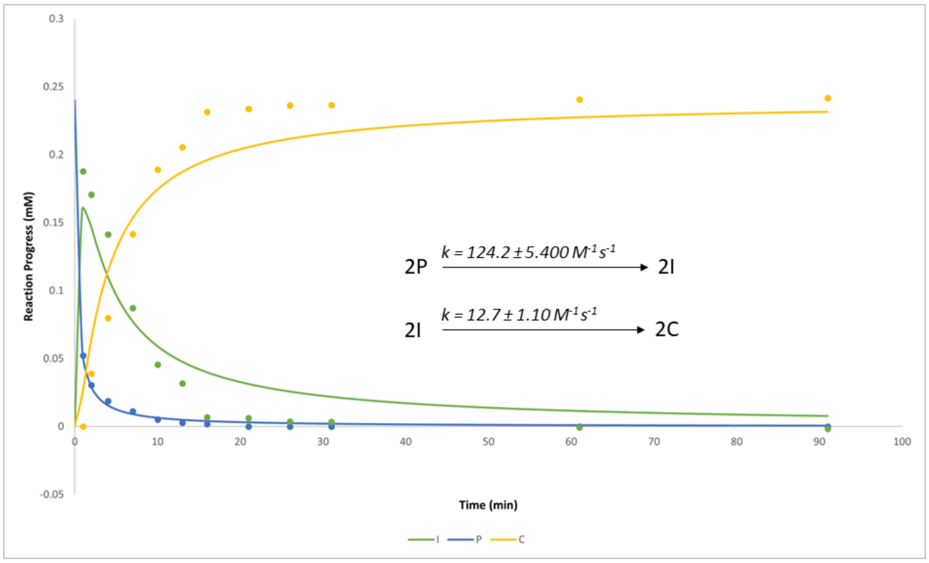

Figure S22: Fitting for $ZrBr_4$: $Zr(O\textit{i}Pr)_4 \cdot \textit{i}PrOH$ reaction data with mechanism 3 with second-order kinetics in both steps. The root mean square (RMS) value for the fit is 0.00315.

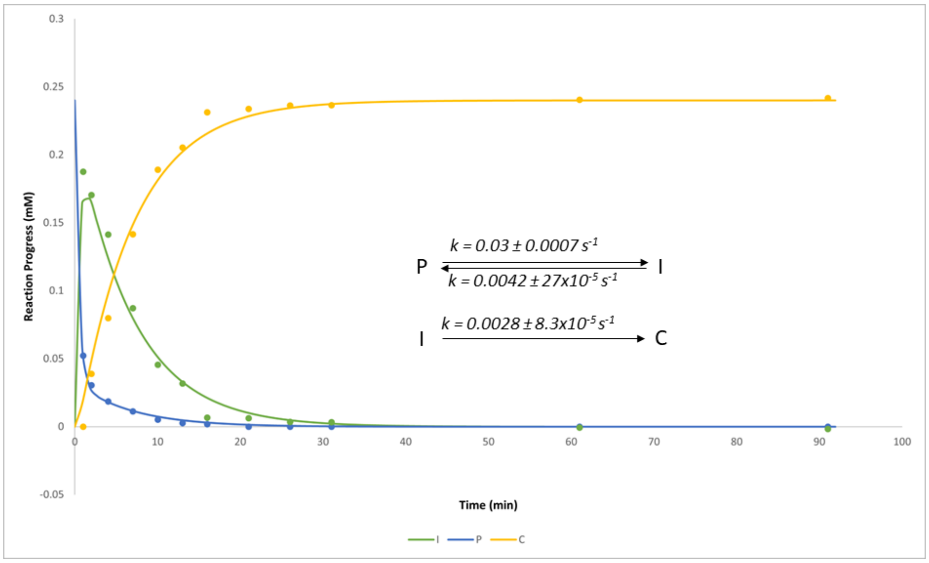

Figure S23: Fitting for $ZrBr_4$: $Zr(O\textit{i}Pr)_4 \cdot \textit{i}PrOH$ reaction data with mechanism 3 with reversible kinetics in step 1. The root mean square (RMS) value for the fit is 0.00150.

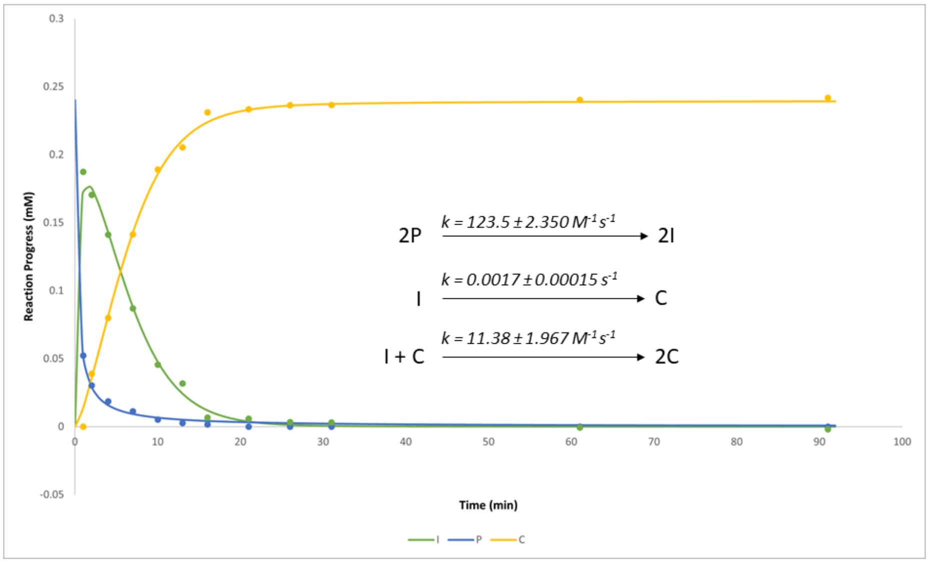

Figure S24: Fitting for $ZrBr_4$: $Zr(O\textit{i}Pr)_4 \cdot \textit{i}PrOH$ reaction data with mechanism 5 with first-order kinetics in step 1. The root mean square (RMS) value for the fit is 0.00137.

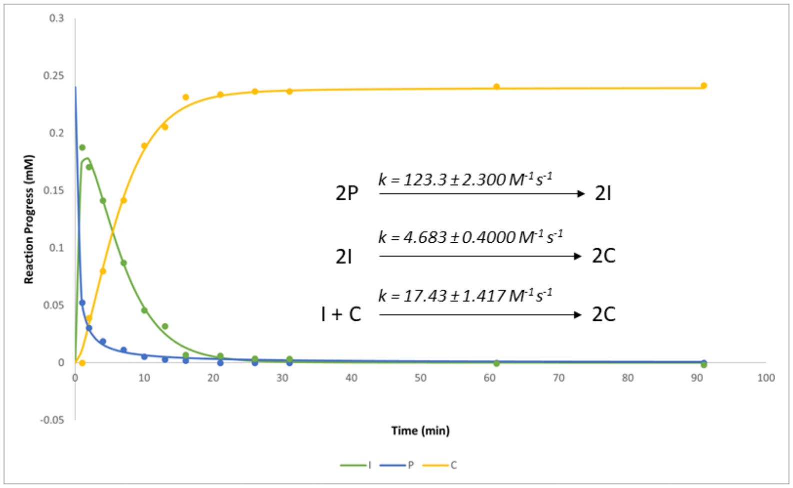

Figure S25: Fitting for $ZrBr_4$: $Zr(O\textit{i}Pr)_4 \cdot \textit{i}PrOH$ reaction data with mechanism 5. The root mean square (RMS) value for the fit is 0.00134.

Size and concentration tuning

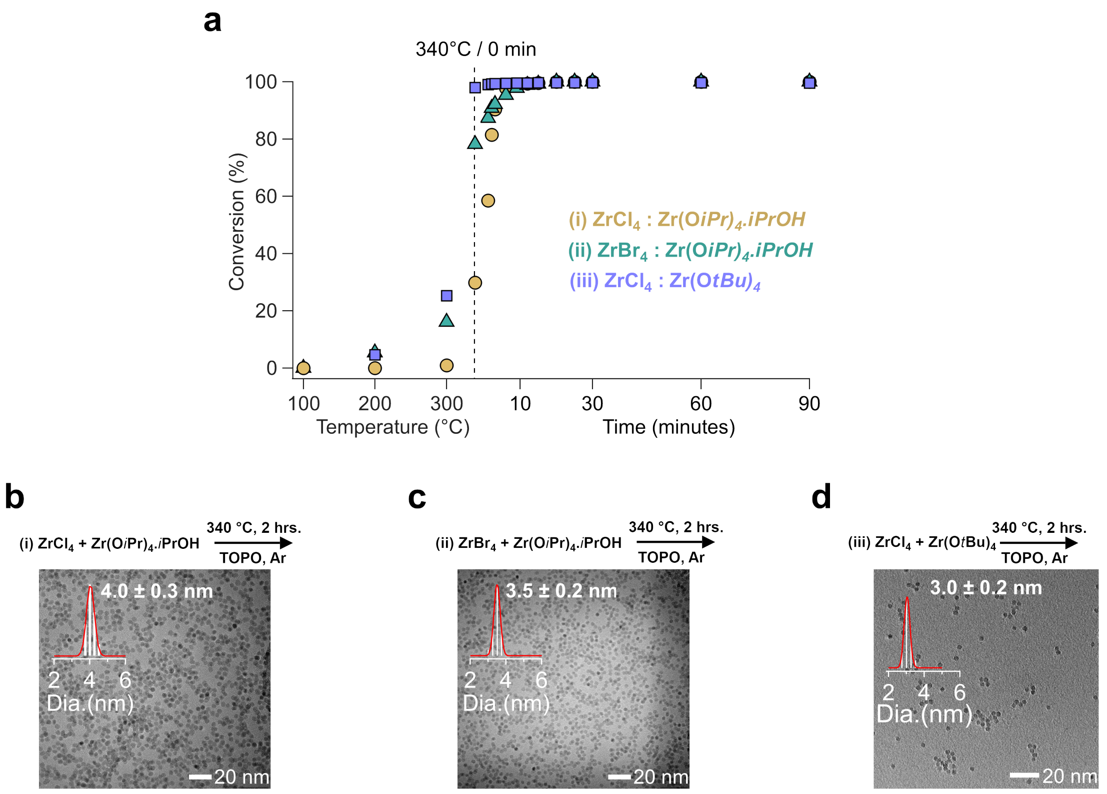

Figure S26: Correlation between precursor conversion rate and final crystal size. (**a**) The precursor conversion trend in three different precursor combinations. TEM image and histogram of purified nanocrystals (**b**) $ZrCl_4$: $Zr(O\textit{i}Pr)_4 \cdot \textit{i}PrOH$, (**c**) $ZrBr_4$: $Zr(O\textit{i}Pr)_4 \cdot \textit{i}PrOH$ and (**d**) $ZrCl_4$: $Zr(O\textit{t}Bu)_4$.

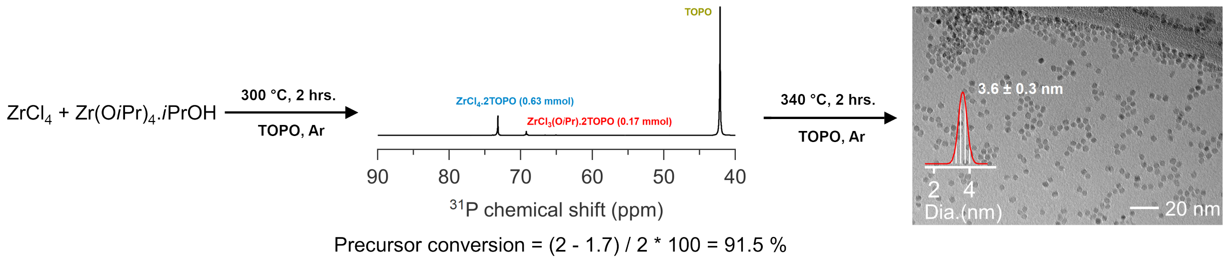

Figure S27: Modified $ZrCl_4$ reaction to reproduce conditions of faster precursor conversion. The modified $ZrCl_4$: $Zr(O\textit{i}Pr)_4 \cdot \textit{i}PrOH$ reaction. The ^{31}P NMR spectrum showing the extent of precursor conversion and TEM image of purified nanocrystals are shown.

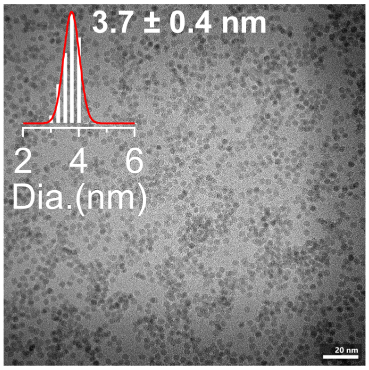

$ZrCl_4$ + $ZrBr_4$

Figure S28: TEM image and histogram of purified nanocrystals synthesized with a mixture of $ZrCl_4$ and $ZrBr_4$ (keeping the total amount of zirconium halide always constant).

References

(S1) Kotlarchyk, M.; Stephens, R. B.; Huang, J. S. Study of Schultz distribution to model polydispersity of microemulsion droplets. *The Journal of Physical Chemistry* **1988**, *92*, 1533–1538.

(S2) Hoops, S.; Sahle, S.; Gauges, R.; Lee, C.; Pahle, J.; Simus, N.; Singhal, M.; Xu, L.; Mendes, P.; Kummer, U. OCOPASI: a COmplex PAthway SImulator. *Bioinformatics* **2006**, *22*, 3067–3074.
